# Optical Nanosensor Passivation Enables Highly Sensitive Detection of the Inflammatory Cytokine Interleukin-6

**DOI:** 10.1021/acsami.4c02711

**Published:** 2024-05-15

**Authors:** Pooja Gaikwad, Nazifa Rahman, Rooshi Parikh, Jalen Crespo, Zachary Cohen, Ryan M. Williams

**Affiliations:** † Department of Biomedical Engineering, The City College of New York, New York, New York 10031, United States of America; ‡ PhD Program in Chemistry, The Graduate Center of The City University of New York, New York, New York 10016, United States of America

**Keywords:** inflammatory cytokines, optical sensors, single-walled carbon nanotubes, SWCNT, clinical diagnostics

## Abstract

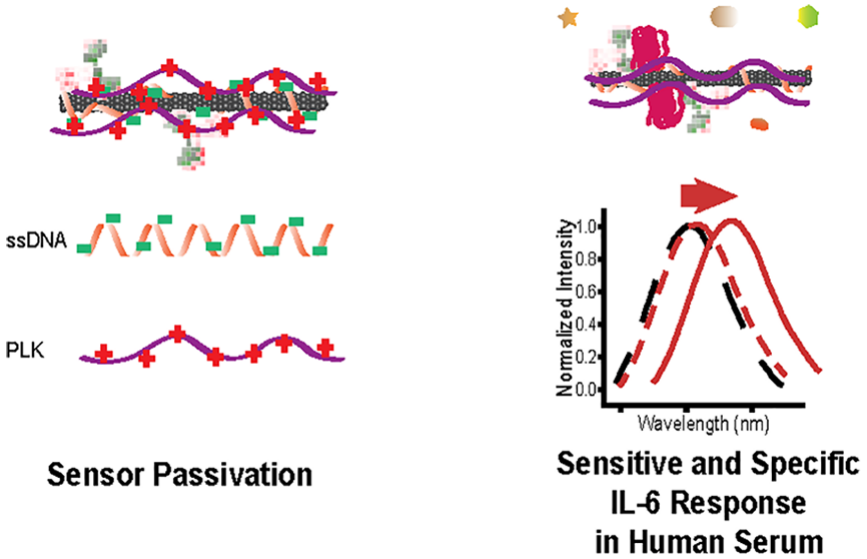

Interleukin-6 (IL-6) is known to play a critical role in the progression of inflammatory diseases such as cardiovascular disease, cancer, sepsis, viral infection, neurological disease, and autoimmune diseases. Emerging diagnostic and prognostic tools, such as optical nanosensors, experience challenges in translation to the clinic in part due to protein corona formation, dampening their selectivity and sensitivity. To address this problem, we explored the rational screening of several classes of biomolecules to be employed as agents in noncovalent surface passivation as a strategy to screen interference from nonspecific proteins. Findings from this screening were applied to the detection of IL-6 by a fluorescent-antibody-conjugated single-walled carbon nanotube (SWCNT)-based nanosensor. The IL-6 nanosensor exhibited highly sensitive and specific detection after passivation with a polymer, poly-l-lysine, as demonstrated by IL-6 detection in human serum within a clinically relevant range of 25 to 25,000 pg/mL, exhibiting a limit of detection over 3 orders of magnitude lower than prior antibody-conjugated SWCNT sensors. This work holds potential for the rapid and highly sensitive detection of IL-6 in clinical settings with future application to other cytokines or disease-specific biomarkers.

## Introduction

Nanobiosensors have substantial potential in medical diagnostics.^1^ Single-walled carbon nanotube (SWCNT)-based optical nanosensors have garnered interest due to their unique near-infrared SWCNT photoluminescence, very little of which is absorbed by biological fluids and tissues.^2−5^ Single-walled carbon nanotubes exhibit a range of chiralities described by (n, m) indices, each having discrete and narrow absorption and fluorescence emission bands, as well as large Stokes shifts.^6−8^ Furthermore, the fluorescence of SWCNT does not decrease or photobleach over time due to excitation, allowing for long-term and frequent imaging and interrogation.^9^

The optoelectronic properties of SWCNT are affected by the surrounding environment, which makes them suitable for biosensing applications. Single-stranded DNA (ssDNA)-wrapped SWCNTs have been used as optical nanosensors for cancers and metabolic diseases, among others.^10−12^ These optical nanosensors allow rapid and inexpensive detection compared to traditional techniques such as immunoassays, mass spectrometry, and others.^13,14^ For example, simultaneous detection in human serum of multiple biomarkers for hepatocellular carcinoma, alpha fetoprotein, and Golgi protein 73, has been reported.^15^ Antibody-conjugated SWCNT-based optical sensors for cardiovascular disease biomarkers,^14^ atherosclerosis,^16^ cancer,^4,17^ diabetes,^18^ and recently COVID-19^19^ have also been reported. Furthermore, successful in vivo detection of protein, lipid, nucleic acid, and small molecule analytes has been demonstrated, for example, human epididymis protein 4, an FDA approved biomarker for high-grade serous ovarian carcinoma,^3^ among others.^20−23^

Interleukin-6 (IL-6) is known to play a critical role in a wide range of diseases such as cardiovascular diseases,^24,25^ viral infections such as COVID,^26^ cancer,^27^ sepsis, and bacterial infection,^28^ neurological diseases,^28^ and autoimmune diseases.^29^ Therapies which reduce the effects of IL6 have significantly improved treatment outcomes for rheumatoid arthritis and COVID-19.^26,30^ The timely determination of elevated IL-6 levels offers clinicians an opportunity to identify individuals at risk for poor outcomes and treat them appropriately. Standard techniques for detection of IL-6 are immunoassays, Western blotting, mass spectroscopy, flow cytometry,^31^ and semiquantitative immunohistochemical detection.^32^ Of these, immunosorbent assays offer the lowest limits of IL-6 detection in human plasma, as low as 0.5 pg/mL.^33,34^ However, these techniques require long incubation periods, trained personnel, and proper instrumentation, making them incompatible for routine clinical diagnosis, frequent testing, and monitoring.^35,36^ To mitigate these limitations, immunosensing antibody-based nanosensors have emerged as a user-friendly point-of-care alternative.^35^ For example, nanosensors for IL-6 based on silica nanowires and metal nanoparticles have been reported.^37−40^

To successfully implement the clinical use of SWCNT-based nanosensors, their sensitivity and selectivity must be retained in complex biological fluids. The formation of a heterogeneous protein corona on the nanotube surface in physiologically relevant environments limits this potential.^20,41−43^ This phenomenon is due to the hydrophobicity of nanotubes, leading to adsorption of proteins and other biologics in a noncovalent and nonspecific manner.^44,45^ This leads to a reorientation of the dipole moments and charge transfer around ssDNA-SWCNTs, leading to photoluminescence modulation.^46^ Therefore, in order to prevent nonspecific nanosensor responses to the detriment of selectivity and sensitivity, it is necessary to prevent such interactions.^10^

As nonspecific interactions with serum components are entropically favored, these coronas are difficult to remove once formed. However, it is possible to saturate the nanotube surface with a homogeneous, known corona prior to nanotube interaction with serum components. Noncovalent surface adsorption prior to sensor deployment, here referred to as passivation, by biocompatible molecules is a widely used strategy for controlling the surface coronas of materials, including nanosensors and standard molecular biology assays such as immunohistochemistry, Western blotting, and immunoassays.^3,4,47,48^ Such strategies are particularly prevalent in assays involving antibodies as molecular recognition elements.

Serum albumin and polyethylene glycol (PEG)-modified phospholipids were used in previous studies as passivation agents to improve the performance of SWCNT-based optical nanosensors.^3,4,10,49^ Proteins such as bovine serum albumin (BSA) and nonfat dry milk (NFDM) have been used extensively in biological assays for passivation due to their low cost and biocompatibility.^50^ PEG-modified phospholipids exhibit unique biomimetic properties and have been used particularly for in vivo applications.^51,52^ Polymers such as polyethylene imine^53^ (PEI), chlorin e6,^54^ and poly-l-lysine^55^ (PLK) have been used to noncovalently adsorb to nanotube surfaces for imaging, rather than sensing, applications.

Here, we studied the efficacy of passivation agents in improving the performance of an IL-6 specific photoluminescent nanosensor in human serum. We screened 8 passivation agents across the general classes of proteins, polymers, and PEG-modified phospholipids for their potential to block nonspecific adsorption of serum components onto the nanotube surface. Promising passivation molecules were then used in the improvement of an antibody-conjugated IL-6 nanotube-based optical sensor in human serum. We found that BSA and PLK, which have not previously been explored for nanosensors, impart pg/mL range detection of IL-6 in this complex biological environment.

## Experimental Section

### Preparation of SWCNT-ssDNA

We initially sought to evaluate the potential of passivation agents using the basic nanotube construct, without a molecular recognition moiety, in order to simplify the screening system. High-pressure carbon monoxide (HiPCO)-prepared single-walled carbon nanotubes (SWCNT) (NanoIntegris Technologies, Inc.; Quebec, Canada) and a single-stranded DNA of the sequence (TAT)_6_ (Integrated DNA Technologies; Iowa, USA) in a 1:2 mass ratio were suspended in 0.5 mL of 1× phosphate buffered saline (Sigma-Aldrich; Missouri, USA). The suspension was sonicated for 1 h at 40% amplitude while on ice via a 120 W ultrasonicator with 1/8 in. probe microtip (Fisher Scientific; New Hampshire, USA). The sonicated suspension was ultracentrifuged for 1 h at 58,000*g* in 4 mL polycarbonate centrifuge tubes (Beckman Coulter; California, USA) using an Optima Max-XP Ultracentrifuge (Beckman Coulter) fit with an MLA-50 rotor. After ultracentrifugation, the top 75% of the suspension was collected and filtered on the day of use with 100 kDa Amicon Ultra 0.5 mL centrifugal filters (Sigma-Aldrich) at 14,000*g* for 15 min to remove unwrapped ssDNA. After filtration, the solution retained in the filter containing ssDNA-SWCNT was washed two times with 200 μL 1X phosphate buffered saline (PBS) and centrifugal-filtered again. Finally, the solution containing SWCNT-(TAT)_6_ retained in the filter was suspended in total 200 μL of 1× PBS.

The concentration of SWCNT-(TAT)_6_ was determined by using a V-730 UV–visible absorption spectrophotometer (Jasco Inc.; Maryland, USA). SWCNT-(TAT)_6_ solution was diluted with 1× PBS to obtain absorbance in the range of 0.3 to 0.7. The concentration of SWCNT was calculated using the value of the absorbance minima near 630 nm (extinction coefficient = 0.036 L mg^–1^ cm^–1^).^3,4,14^

### Fluorescence Spectroscopy for Screening

Near-infrared fluorescence emission spectra of SWCNT-(TAT)_6_ were acquired with a MiniTracer spectrophotometer (Applied NanoFluorescence; Texas, USA). The laser source has an excitation wavelength of 638 nm and emission spectra were obtained between 900 and 1400 nm. SWCNT (7,5), (7,6), and (9,5) chiralities were analyzed using custom MATLAB code (available upon request) which fit emission peaks to a baseline-subtracted pseudo-Voigt model.^3,4,56^ Fits were ensured to have goodness of fit values (R^2^) of greater than 0.98 prior to analyses. Center wavelengths were obtained from each fit, as well as maximum intensity values.

### Screening of Passivation Agents in Complex Biological Media

Passivation of SWCNT-(TAT)_6_ complexes was achieved by incubating 0.5 mg/L SWCNT-(TAT)_6_ and passivating agents dissolved in 1× PBS in the desired mass ratio at 4 °C for 30 min. Passivation agents used, including BSA, NFDM, casein, PEG-1500, PEI, PLK, 16:0 PE PEG, and DSPE-PEG-amine (Table 1; Figure S1), were evaluated in mass ratios of 5×, 25×, 50×, and 100× greater than the nanotube complex. 10% heat inactivated fetal bovine serum (FBS) (Corning; New York, USA) was used to challenge each passivation, simulating complex in vivo biological conditions. Changes resulting from the addition of FBS to passivated SWCNT-(TAT)_6_ were quantified by a change in the fluorescence emission center wavelength for the (7,5), (7,6), and (9,5) chiralities. Data were collected at 2, 15, 30, 60, 120, 150, and 180 min after challenge with FBS and acquired in triplicate.

**Table 1:** Passivation Agents Used in Screening

Class	Passivating Agents
Proteins	• Bovine serum albumin (BSA), heat-inactivated: Fisher Scientific (New Hampshire, USA)
	• Nonfat dry milk (NFDM), powder: Santa Cruz Biotechnology (Texas, USA)
	• Casein: EMD Millipore (Massachusetts, USA)
Polymers	• Polyethylene glycol (PEG), MW = 1500, Ω-end and α-end with hydroxyl group: Sigma-Aldrich (Missouri, USA)
	• Polyethylene imine (PEI), MW = 10,000: Alfa-Aesar (Massachusetts, USA), Branched
	• Poly-l-Lysine (PLK), MW = 70,000 to 150,000 Da: Advanced Biomatrix (California, USA)
Phospholipids	• Ammonium salt of 1,2-dipalmitoyl-sn-glycero-3-phosphoethanolamine-N-[methoxy(polyethylene glycol)-2000] (16:0 PE PEG), FW = 2749.42: Avanti Polar Lipids Inc. (Alabama, USA)
	• 1,2-distearoyl-sn-glycero-3-phosphoethanolamine-N-[amino (polyethylene glycol)-2000] (DSPE-PEG-amine), MW = 2000: BroadPharm (California, USA)

### Absorption Spectroscopy

Near-IR absorption spectra in the range of 900–1400 nm were acquired using a MiniTracer spectrophotometer to analyze the formation of stable interactions between passivation agents and SWCNT constructs. Data were analyzed using custom MATLAB code as described above. Mass ratios of 5×, 10×, 25×, and 50× of the passivation agent in comparison to 10 mg/L SWCNT-(TAT)_6_ were used. Changes in absorbance resulting from nanotube passivation were quantified by changes in the absorbance center wavelength peaks of nanotube chiralities (7,5), (7,6), (9,5), and (6,5). Data were collected at 2, 15, 30, 60, 120, 150, and 180 min postpassivation to confirm the duration for which passivation is stable. Data were acquired in triplicate.

### IL-6 Sensor Synthesis

SWCNT-(TAT)_6_ was prepared as above with the modification that the ssDNA sequence contained a 3′ primary amine modification (5′-(TAT)6)/3AmMO/-3′) (Integrated DNA Technologies). The amine-modified ssDNA-SWCNT complex was conjugated to a monoclonal IL-6 antibody (Catalog number 554543; BD Biosciences, California, USA) using carbodiimide conjugation chemistry similarly to previous studies.^3,4^ Briefly, the carboxylic acid group of the antibodies was activated using 1-ethyl-3-(3-dimethylainopropyl)carbodiimide) (Sigma-Aldrich) and N-hydroxysuccinimide (TCI Chemicals, Oregon, USA), in a 10× and 25× molar excess, respectively, for 15 min at 4 °C. The reaction was quenched with 1 μL of 2-mercaptoethanol (Sigma-Aldrich). The activated antibodies were mixed with SWCNT-(TAT)_6_-NH_2_ in 1:1 molar ratio of ssDNA to antibody. The reaction mixture was incubated at 4 °C on ice for a total of 2 h, with gentle brief vortexing every 30 min. The reaction mixture was dialyzed against deionized water with a 1,000 kDa molecular weight cutoff filter (Float-A-Lyzer G2; Spectrum Labs, California, USA) at 4 °C for 48 h with two dialysate changes.

### Physicochemical Characterization of the IL-6 Nanosensor

To confirm the successful conjugation of antibody to the ssDNA-nanotube construct, we performed light scattering measurements. Dynamic light scattering (DLS) was performed (Nano-ZS90, Malvern: Worcestershire, United Kingdom) for nanosensor and SWCNT-(TAT)_6_-NH_2_ nanotube complexes as previously described to determine their relative sizes.^3,4^ Electrophoretic light scattering (ELS) (Nano-ZS90, Malvern) was performed to compare the relative ζ potential of the nanosensor and SWCNT-(TAT)_6_-NH_2_ complex. Data were acquired in triplicate.

### Evaluation of IL-6 Nanosensor Function

To confirm basic functionality of the IL-6 nanosensor complex, we first evaluated the fluorescence response of 0.5 mg/L nanosensor to 5250 ng/mL human IL-6 (Catalog number RP8619; Thermo Fisher Scientific, Massachusetts, USA) in 1× PBS using the MiniTracer as described above with identical time points and data processing. The response of the sensor passivated with 50× BSA as in prior studies was analyzed in 10% Fetal Bovine Serum as described above. All experiments were performed in triplicate.

### IL-6 Nanosensor in Human Serum

The 0.5 mg/L IL-6 nanosensor was passivated with 50× mass ratios of PLK, NFDM, and BSA at 4 °C for 30 min. Fluorescence data were acquired before and after adding pooled human serum (MP Biomedicals; California, USA) with or without spiked IL-6 using a custom-built high-throughput near-IR plate reader spectrophotometer (ClaIR, Photon Etc.; Montreal, Canada). Spiked IL-6 protein was added to final concentrations of 25, 250, 2500, and 25,000 pg/mL. Control responses were obtained with the nanosensor construct without passivation. Data were acquired in triplicate. Custom MATLAB code was used to analyze and fit individual nanotube chirality peaks to a pseudo-Voigt model (code is available upon request). The analyzed fluorescence emission chiralities were (7,5), (7,6), and (9,5) for excitation with the 655 nm laser source and (10,2), (9,4), (8,6), and (8,7) for excitation with the 730 nm laser source. Center wavelength shifts were calculated and compared to those of passivated sensors in human serum with no IL-6 added.

### Statistical Analysis and Data Processing

All statistical analyses were performed in OriginPro 2021 (OriginLab Corporation; Massachusetts, USA). Passivation agent screening experiments were analyzed by one-way analysis of variance (ANOVA) with Dunnett posthoc analysis. P-values were assigned ****P < 0.0001, ***P < 0.001, **P < 0.01, and *P < 0.05. Zeta potential results for IL-6 nanosensor characterization and in vitro performance of the IL-6 nanosensor were analyzed by a two-tailed *t* test. Experiments in human serum were analyzed via one way ANOVA with Dunnet posthoc analysis to compare to the nonpassivated control. Changes in SWCNT center wavelength were reported relative to their emission prior to analyte addition. Custom MATLAB code was used for data processing, including peak fitting to a pseudo-Voigt model. The center wavelengths reported were obtained through the peak fitting and code used for analysis is available upon request.

## Results and Discussion

### Interference Caused by Nonspecific Proteins to Nanosensors

**Figure 1:**
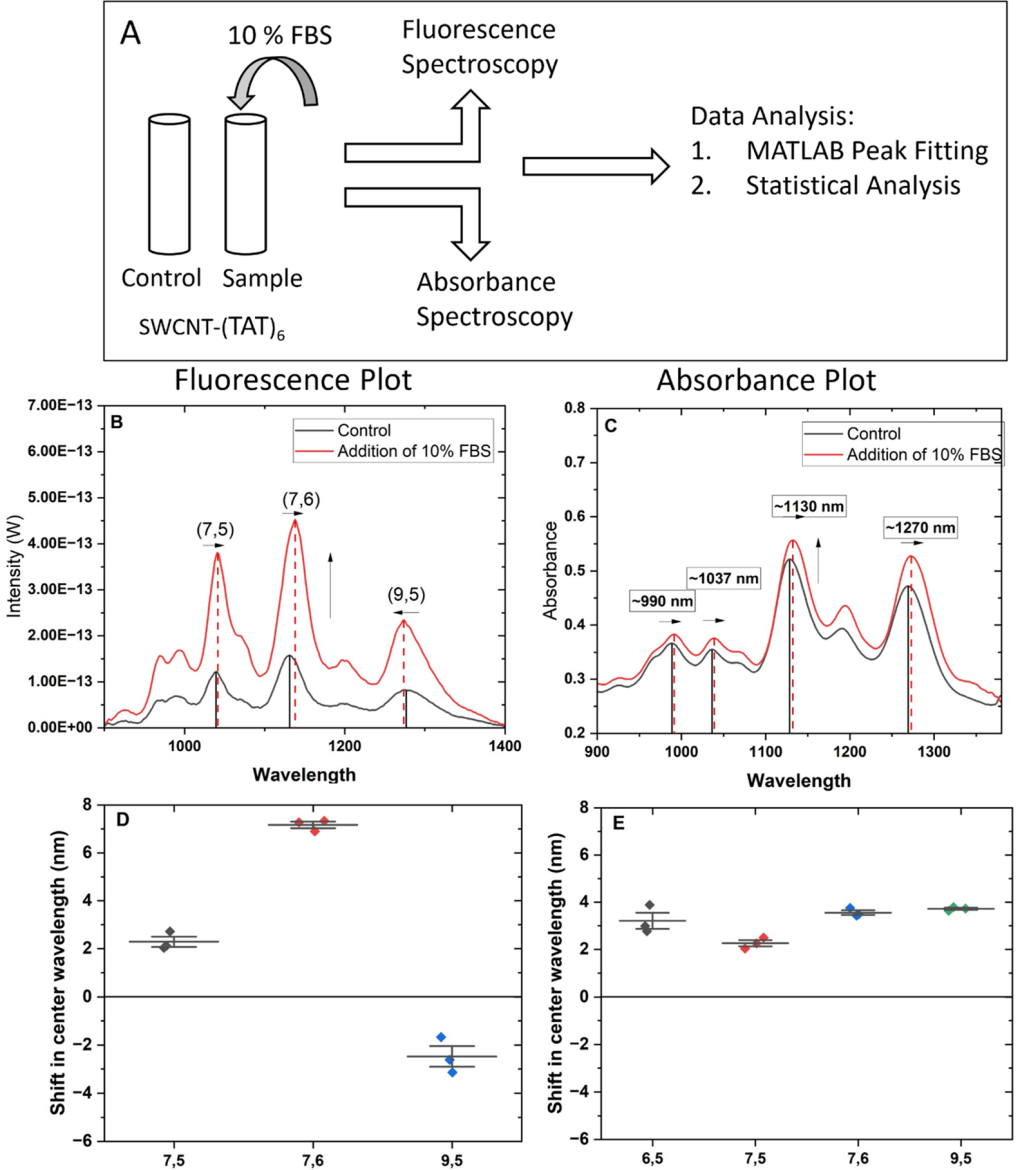
Nonspecific interactions of 10% FBS with SWCNT-(TAT)_6_. (A) Schematic representation of experimental design. (B) In the presence of 10% FBS, the fluorescence intensity of SWCNT-(TAT)_6_ increases by at least 2.5 times across the (7,5), (7,6), and (9,5) chiralities. These chiralities also show blue and red shifts in the presence of 10% FBS. (C) The absorbance value of SWCNT-(TAT)_6_ increases by approximately 10% upon addition of 10% FBS. It also results in a red shift of the center wavelength of ∼990 nm, 1035 nm, 1130 nm, and 1270 nm. (D) Shift in center wavelength of the E_11_ fluorescence peaks for chiralities (7,5), (7,6), and (9,5), *n* = 3. (E) Shift in center wavelength of the E_11_ absorbance peak for chiralities ∼990, 1035, 1130, and 1270 nm, *n* = 3.

Fetal bovine serum (FBS) is commonly used in sensor development studies as a model complex protein environment.^57^ We assessed the nonspecific interactions of FBS with SWCNT-(TAT)_6_ by evaluating shifts in the center wavelength of the E_11_ peaks in absorbance and fluorescence spectra (Figure 1A–C). Fluorescence peaks exhibited substantial shifts in the presence of 10% FBS. The shifts in the (7,5), (7,6), and (9,5) chiralities were, respectively, red-shifted (bathochromic) 2.3 ± 0.37, 7.2 ± 0.24, and blue-shifted (hypsochromic) 2.5 ± 0.7 nm (Figure 1D). Absorbance peaks exhibited center wavelength shifts of 3.22 ± 0.6, 2.3 ± 0.23, 3.6 ± 0.2, and 3.7 ± 0.08 nm for (6,5), (7,5), (7,6), and (9,5) nanotube chiralities, respectively (Figure 1E). We hypothesized that passivation agents which can reduce the magnitude of photoluminescence changes upon challenging with 10% FBS would improve the sensitivity and specificity of physiologically relevant nanosensors.

### Proteins as Passivating Agents

**Figure 2:**
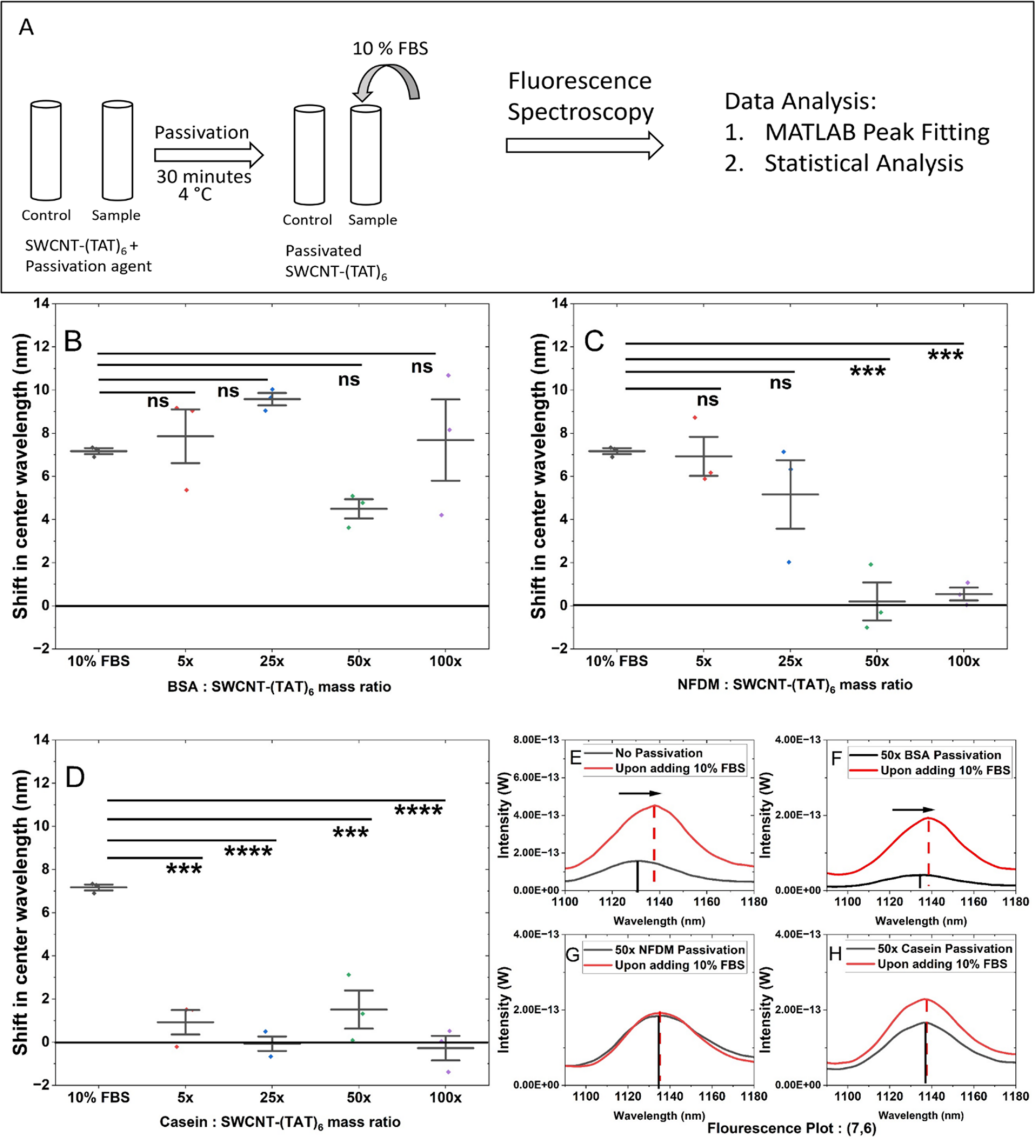
Screening serum interference with protein passivation of SWCNT-(TAT)_6_. (A) Schematic of the experimental design. Shifts in emission center wavelength for (7,6) upon adding FBS (B) for BSA passivation, *n* = 3, all mass ratios demonstrate no statistically significant shift compared to the interference of 10% FBS (C) for NFDM passivation, *n* = 3, 50× mass and 100× mass ratio show minimal shift in spite of addition of 10% FBS, indicating successful screening effect against FBS. (D) for Casein passivation, *n* = 3, all mass ratios show minimal shift upon addition of 10% FBS indicating screening effect. Comparison of emission center wavelength of (7,6) chirality: (E) in presence and absence of 10% FBS for nonpassivated SWCNT-(TAT)_6_. (F) 50× mass ratio BSA passivation and upon challenging it with 10% FBS. (G) 50× mass ratio of NFDM passivation and (H) 50× casein passivation, both showing no change in center wavelength, indicating a successful screening effect.

Previous studies have shown that BSA successfully screened interference caused by nonspecific proteins and improved selectivity of the nanosensors.^3,4^ Here, we explored higher and lower mass ratios compared to the previously used 50× mass ratio (Figure 2A). Three hours after challenging with FBS, we observed a shift of 7.9 ± 2.2 nm for 5×, 9.6 ± 0.5 nm for 25×, 4.6 ± 0.26 nm for 50×, and 7.7 ± 3.3 nm for 100× for the (7,6) fluorescence peak (Figure 2B). Across all ratios, upon challenge with FBS, BSA passivation did not significantly mitigate FBS-induced shifting. Similar observations were made for the (7,5) and (9,5) fluorescence peaks (Figures S2–S6).

We further explored other protein passivation agents, including NFDM and its primary component, casein protein. We found that NFDM passivation mitigated shifting at the 50× mass ratio (Figure 2C). We observed that a 50× mass ratio was optimal for NFDM and casein passivation, with shifts of 0.2 ± 1.5 nm (Figure 2C) and 1.5 ± 1.5 nm (Figure 2D), respectively, for the (7,6) chirality. A 50× mass ratio was optimal for interference screening of the (7,5) and (9,5) chiralities as well (Figures S2, S3). For casein passivation, as low as a 5× mass ratio was found to be effective for all three chiralities investigated (Figure 2D; Figures S2, S3), whereas NFDM passivation was not effective at this ratio (Figure 2C; Figures S2, S3). As casein is just one component of NFDM,^58^ this may contribute to the difference in effectiveness at various mass ratios. The response for all three observed chiralities remained relatively stable for 180 min in the presence of each passivation agent (Figures S7, S8, and S9).

### Polymers as Passivation Agents

We next investigated several polymers as potential passivating agents. We evaluated two cationic polymers, PLK and PEI, and one anionic polymer, 1500 Da PEG (Figure 2A). PEI and PLK have been used to noncovalently wrap SWCNTs for various applications, though not as passivating agents.^53,55^ PEG has been used to covalently functionalize SWCNTs and was chosen to help us distinguish the effect of charge-based interactions on the ssDNA-wrapped SWCNTs. We hypothesized that cationic polymers would be better candidates for passivation due to attractive ionic interactions with anionic ssDNA.

**Figure 3:**
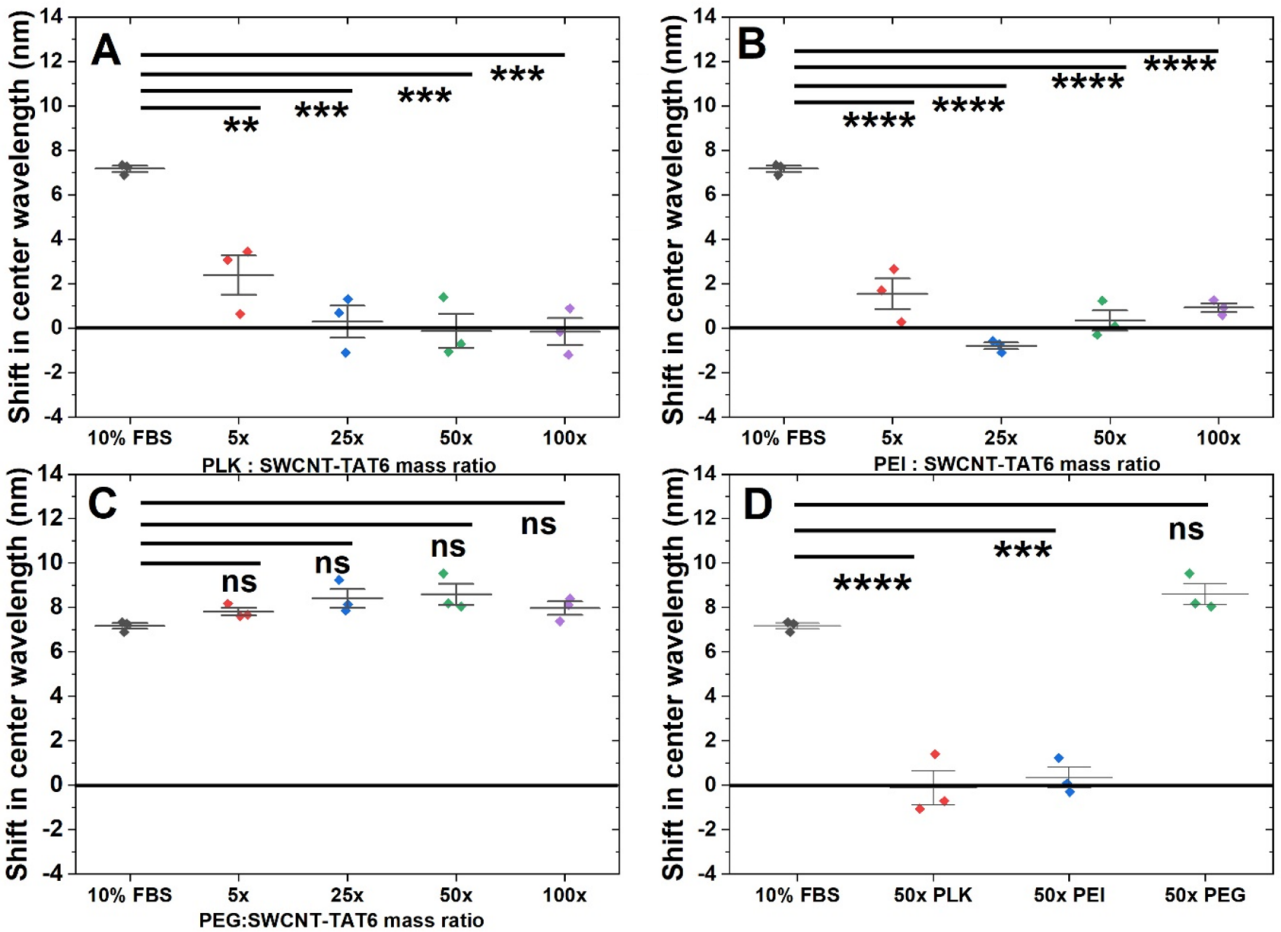
Screening serum interference with polymer passivation of SWCNT-(TAT)_6_. Shift in emission center wavelength for (7,6) upon adding FBS (A) for PLK passivation and (B) for PEI passivation *n* = 3, all mass ratios show minimal shift upon challenging with 10% FBS. (C) for PEG passivation, *n* = 3, shift due to interference of 10% FBS is observed for all mass ratios, indicating poor screening effect by this passivation agent. (D) Comparison of passivation agents at a 50× mass ratio, shift in emission center wavelength *n* = 3, mean ± SD. FBS and 50× PLK (7.3 nm, *p* = 6.6 × 10^–5^); FBS and 50× PEI (6.8 nm, *p* = 1.5 × 10^–4^); FBS and 50× PEG (1.4 nm, *p* = 0.24).

PLK demonstrated a decrease in FBS interference across all passivation ratios investigated (Figure 3A). Branched 10 kDa PEI demonstrated a decrease in interference caused by FBS for all ratios investigated (Figure 3B), although significant visible flocculation of the nanotube construct was observed (Figure S10). It is likely that this results from the branched nature of the PEI used, with multiple amine groups, bringing multiple nanotube constructs physically closer together. Passivation of the nanotube construct with PLK resulted in minimal shifts and no flocculation at all mass ratios investigated upon challenging with FBS, with all shift magnitudes ≤0.3 nm (Figure 3A; Figures S11, S12). We then evaluated PEG-1500, an anionic polymer, demonstrating no screening effect against FBS at all ratios (Figure 3C; Figures S11, S12). It is likely that the repulsive Coulombic interactions between anionic ssDNA and anionic PEG results in poor interaction with the nanotube surface, which in turn allows FBS components to interact with SWCNT-(TAT)_6_ nonspecifically. PLK passivation showed no significant screening effect for the (9,5) chirality due to high variability, though it did for the (7,6) and (7,5) chiralities, indicating that a given passivation agent may show effective passivation at some, but not all, chiralities (Figure 3A; Figures S11, S12). The response for all three fluorescence peaks for all three passivation agents remained relatively stable for a period of 180 min following addition of FBS as a challenger (Figures S13, S14, and S15).

### Phospholipids as Passivation Agents

**Figure 4:**
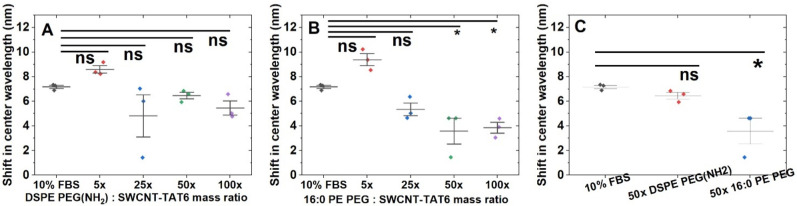
Screening serum interference with phospholipid passivation of SWCNT-(TAT)_6_. Change in emission center wavelength upon challenging with FBS. Shift in emission center wavelength for (7,6) upon adding FBS (A) for DSPE PEG (NH_2_) passivation, *n* = 3, all mass ratios show shift in center wavelength which is statistically similar to that caused by FBS interference (B) for 16:0 PE PEG passivation, *n* = 3, 50× and 100× mass ratios show minimal shift upon challenging with FBS, indicating screening effect by these to mass ratios. (C) Comparison of passivation agents at a 50× mass ratio, shift in emission center wavelength *n* = 3, mean ± SD. FBS and 50× DSPE PEG (NH_2_) (0.7, *p* = 0.7); FBS and 50× 16:0 PE PEG (3.6 nm, *p* = 3.6 × 10^–2^).

We next evaluated two PEGylated phospholipids, 16:0 PE2000PEG and DSPE PEG (NH_2_) (Figure 2A). PEGylated phospholipids are biocompatible and hence well suited for in vivo applications. 16:0 PE2000PEG has been used to successfully passivate SWCNT-based nanosensors and to prepare SWCNT suspensions.^49,52^ All ratios of DSPE PEG (NH_2_) passivation showed no significant change in center wavelength compared to controls for all three chiralities investigated (Figure 4A; Figures S16, S17). For 16:0 PE2000PEG passivation, 50× and 100× mass ratio passivation showed screening of FBS interference for the (7,6) and (9,5) chiralities only (Figure 4B; Figures S16, S17). Again, the responses for most chiralities remained relatively stable for up to 180 min (Figures S18, S19). For agents indicating successful screening of FBS interference, the mass ratios 50× and 100× gave comparable passivation results. Therefore, we concluded that a mass ratio of 50× is optimal for those agents as to not saturate the system with unnecessary components.

We also investigated the effect of passivation on the intensity of SWCNT-(TAT)_6_ (Figure S20). We observed the intensity of SWCNT-(TAT)_6_ before and after passivation, finding that the (7,6) chirality demonstrated an increase in intensity after 50× BSA passivation. 50× NFDM and casein passivation did not substantially impact the intensity. PLK and 16:0 PE PEG 50× passivation did not impact the intensity of SWCNT-(TAT)_6_. We also observed that NFDM, casein, PLK, and 16:0 PE PEG passivation agents had a minimal impact on the intensity of the SWCNT-(TAT)_6_ when compared to 10% FBS.

### Analysis of Corona Formation via Absorbance Spectroscopy

Changes in the proximal environment of SWCNT-(TAT)_6_ due to close interactions and/or homogeneous corona formation with the passivating agents may be reflected by changes in the E_11_ absorbance maxima of each chirality.^59−61^ We hypothesized that the ability of the passivation agent to screen nonspecific interactions is correlated to its ability to form a stable corona around the nanotube complex. We assumed that stronger interactions of a given passivation agent with the nanotube surface would result in greater changes in the center wavelength of the SWCNT absorption peaks. Further, we propose that to successfully screen nonspecific interactions by 10% FBS, the passivation agent should interact with SWCNT-(TAT)_6_ more strongly than FBS itself. Therefore, we anticipated that passivation agents that result in larger absorbance shifts than that caused by 10% FBS would exhibit strong passivation capability. For this reason, in our statistical analysis, changes in individual chirality absorption center wavelengths in the presence of 10% FBS were compared with those in the presence of passivation agents.

**Figure 5:**
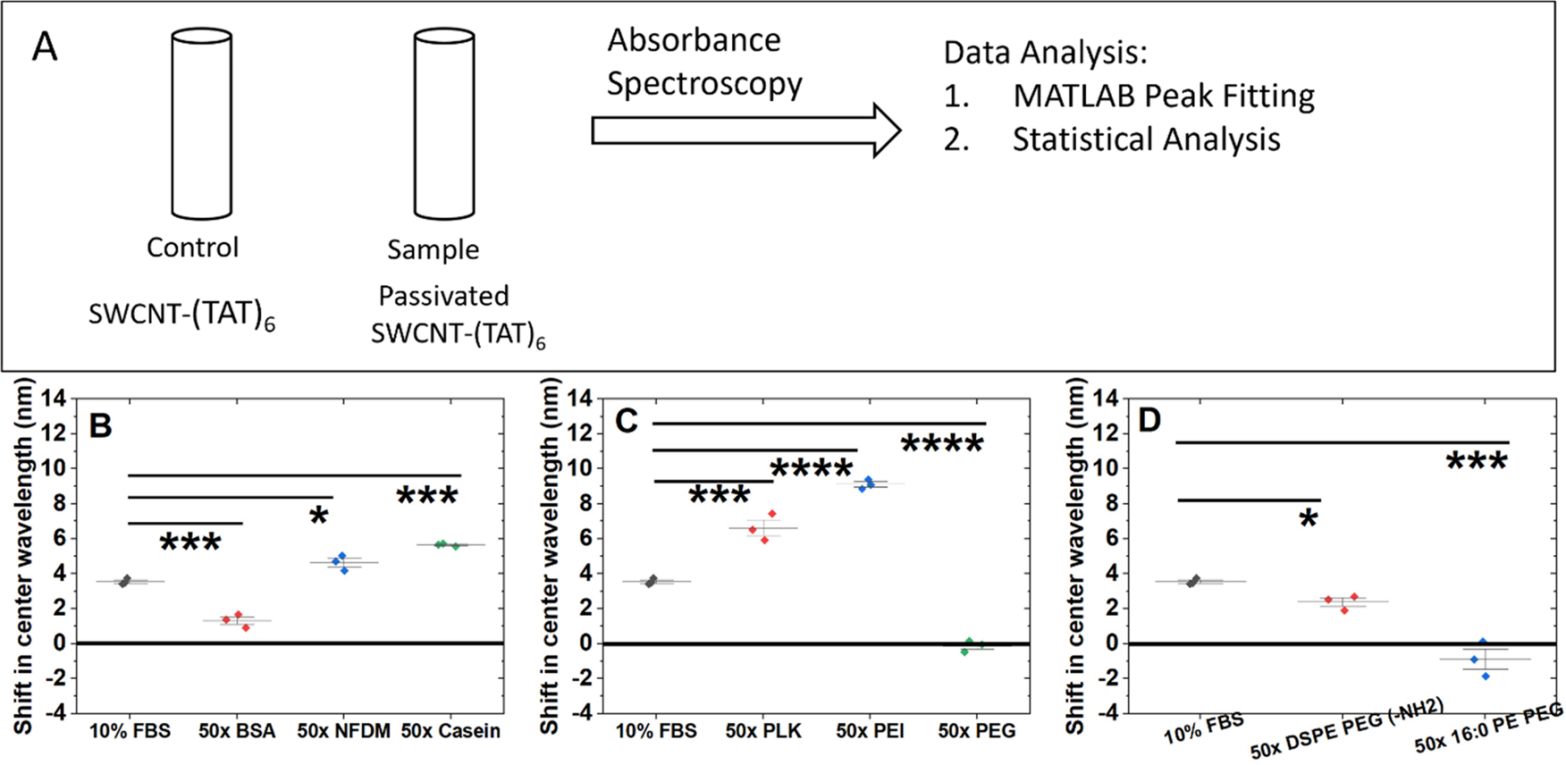
Change in absorbance as a measurement of homogeneous and stable passivation. (A) Schematic of experimental design. Comparison of the ability to influence the proximal environment of SWCNT-(TAT)_6_ for the (7,6) peak by (B) 50× mass ratio for protein passivation. *n* = 3, NFDM and Casein show shift in center wavelength higher than that of FBS. (C) 50× mass ratio polymers. *n* = 3, mean ± SD. PLK and PEI show shifts higher than that of FBS. (D) 50× mass ratio phospholipids, *n* = 3, none of them show shift higher than that of FBS. In absorbance spectra, higher shift by passivation agent indicates stronger interaction with SWCNT-(TAT)_6_. Hence, NFDM, Casein, PLK, and PEI have a higher potential to act as screening agents against FBS as observed independently in Figures 1, 2, and 3.

For nonpassivated SWCNT-(TAT)_6_ in serum conditions, the center wavelength of the (7,6) chirality was shifted by 3.6 ± 0.2 nm as compared to buffer conditions (Figure 1E). We found that, at 180 min postpassivation with 50× BSA, the (7,6) emission peak center wavelength shifted by 1.32 ± 0.4 nm, smaller than 3.6 ± 0.2 nm for 10% FBS (Figure 5B). We observed similar responses for the (7,5), (9,5), and (6,5) chiralities (Figures S21A, S22A, and S23A). A significant shift was observed after addition of 50× NFDM for the (7,6) and (9,5) chiralities of 4.6 ± 0.4 and 4.56 ± 0.35 nm, respectively (Figure 5B; Figure S22A). Further, 50× casein passivation showed a higher shift magnitude of 5.7 ± 0.1 nm than that for FBS alone (Figure 5A).

Of the polymeric passivation agents investigated, 50× PLK and 50× PEI demonstrated absorbance shifts of 6.6 ± 0.8 and 9.6 ± 1 nm, respectively (Figure 5B). A similar trend was observed for these two agents when evaluating the (7,5) and (9,5) chiralities (Figures S21B, S22B). For 50× PEG passivation, the (7,6) absorption center wavelength was shifted by −0.14 ± 0.3 nm (Figure 5B). For all other peaks, 50× PEG demonstrated a consistent blue shift compared to that of serum conditions (Figures S21B, S22B, and S23B).

Upon evaluation of absorption shifts induced by phospholipid agents, 50× DSPE PEG (NH_2_) caused a shift of 2.38 ± 0.4 nm, which is smaller than that of FBS (Figure 5C). For this chirality, 50× 16:0 PE PEG passivation caused a blue shift of 0.9 ± 1 nm (Figure 5C). Other chiralities investigated exhibited similar trends for both phospholipid-based agents (Figures S21C, S22C, and S23C). Time-course measurements up to 180 min demonstrated relative stability during this period (Figures S24, S25, and S26).

It is likely that proteins, such as BSA, NFDM, and casein, may cause specific changes by forming a homogeneous protein corona. These proteins may interact with the nanotube surface through hydrophobic adsorption and/or charge–charge interactions with the ssDNA. Similarly, the interactions between cationic polymers such as PEI and PLK with SWCNT surfaces may be aided by strong Coulombic interactions, allowing a robust and stable corona to form. Further, we propose that the cationic charge and linear structure of PLK may aid in interactions due to the Coulombic attraction with SWCNT-(TAT)_6_ without flocculation as observed for cationic but branched PEI. The PEG construct which we used is anionic and therefore expected to show little interaction due to charge repulsion with the ssDNA. Further, changes in absorbance due to the interaction with 16:0 PE2000PEG are likely due to the hydrophobic interaction of the lipid tails with the SWCNT surface.^62^ DSPE-PEG (NH_2_) is a zwitterionic PEG modified lipid with a long hydrophobic tail,^62^ which may facilitate hydrophobic and/or charge–charge interactions. However, for screening of serum interference, this phospholipid did not exhibit sufficient passivation.

### Construction and Validation of an IL-6 Nanosensor

We then sought to apply the passivation tools developed here for use with a clinically relevant nanosensor device. We prepared and characterized a nanosensor composed of a monoclonal IL-6 antibody conjugated to SWCNT-(TAT)_6_-NH_2_ (Figure S27). Dynamic light scattering (DLS) revealed successful conjugation as the correlation coefficient demonstrated a larger relative particle size for the conjugate nanosensor compared to unconjugated SWCNT-(TAT)_6_-NH_2_ complexes as previously described (Figure S27A).^3,4^ Further, electrophoretic light scattering demonstrated a more negative ζ-potential for SWCNT-(TAT)_6_-NH_2_ than that for the IL-6 nanosensor conjugate, confirming successful antibody conjugation as in prior studies (Figure S27B).^3,4^

We first tested the sensor fluorescence response to 5.25 μg/mL IL-6 in simple buffer conditions of 1× PBS. The center wavelength of the (7,6) chirality of the nanosensor blue-shifted 3.6 ± 0.63 nm (p = 0.02) (Figure S27C). In addition, the (7,5) and (9,5) chiralities demonstrated a statistically significant change in center wavelength (Figure S28A,B).

### Detection of IL-6 in Human Serum

Having validated basic IL-6 nanosensor function, we next evaluated its function in a simulated clinical environment using human serum. To do so, we passivated separate batches of the IL-6 nanosensor with BSA, PLK, and NFDM at a ratio of 50×. We did not choose any phospholipid-PEG species as there was no or only slight benefit in preventing serum-induced shifting (Figure 4), and there was a lower magnitude of absorbance change, signifying a less stable interaction with the ssDNA-SWCNT construct (Figure 5C).

These were chosen as they exhibited substantial screening responses above as well as the formation of a stable homogeneous corona as revealed by absorbance spectroscopy. Serum IL-6 levels in healthy patients typically range from 1 to 10 pg/mL.^25,28^ For patients with health conditions such COVID-19 or sepsis, serum concentrations are elevated, reaching 10,000 pg/mL or greater.^28,63,64^ Considering this, we simulated disease conditions by spiking known quantities of IL-6 into human serum at concentrations of 25; 250; 2,500; and 25,000 pg/mL. We then compared shifts in fluorescence center wavelength for each chirality and determined whether the shifts were statistically significant and consistent compared to controls.

**Figure 6:**
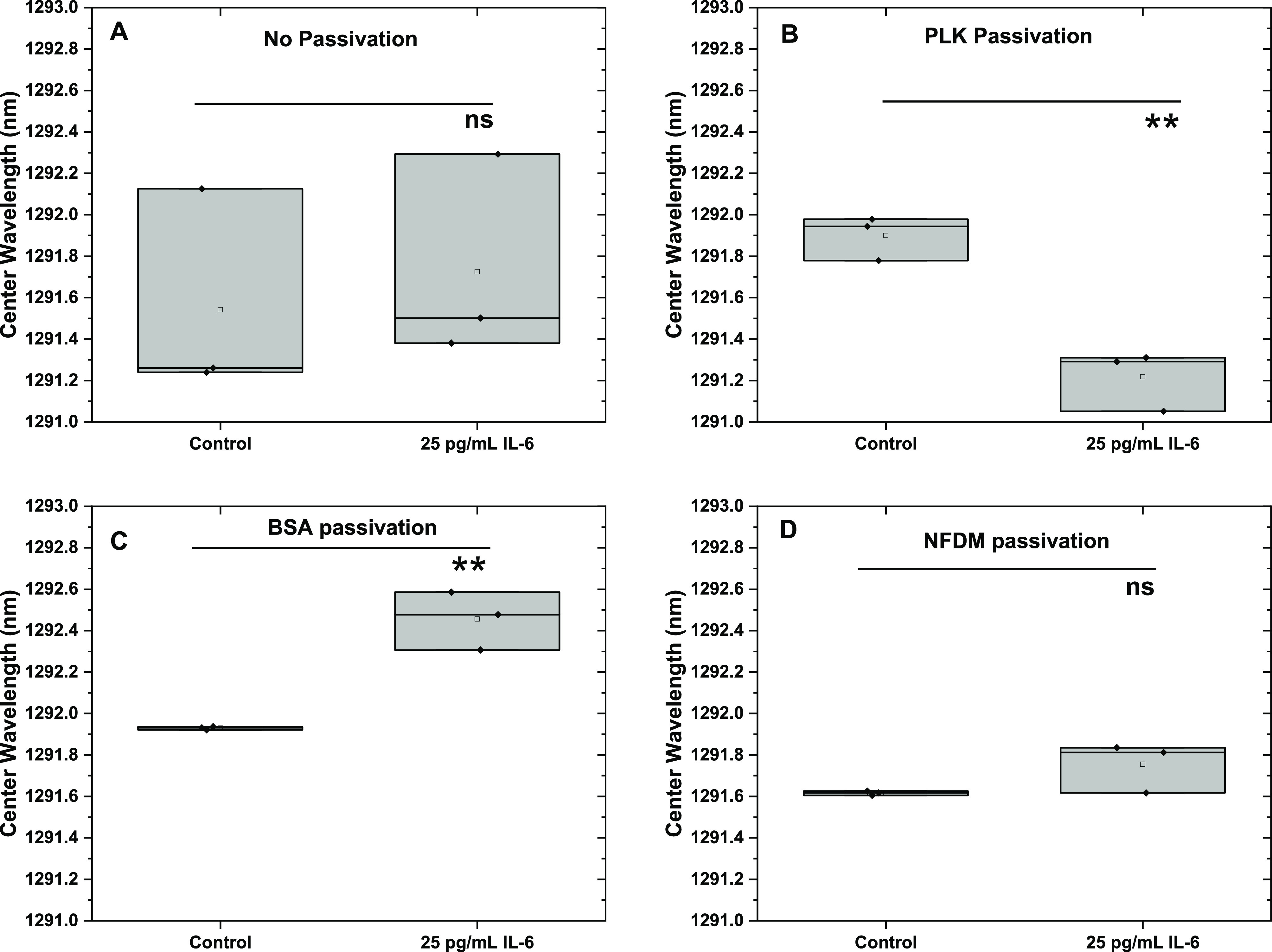
Response of (8,7) chirality IL-6 nanosensor to 25 pg/mL IL-6 spiked in human serum. (A) For the nonpassivated nanosensor, the observed change in the center wavelength was not significant in the presence of IL-6, indicating no detection of IL-6. (B) The PLK passivated nanosensor detected the presence of IL-6 through change in the center wavelength. (C) The BSA passivated nanosensor also demonstrated a significant red-shift, while (D) the NFDM passivated nanosensor did not respond to the presence of IL-6.

We found that, by evaluating four chiralities at once, we were able to positively identify each concentration investigated, including the extremely low and clinically relevant 25 pg/mL. Of the three passivation agents evaluated, we observed that the PLK passivated (8,7) chirality demonstrated a significant shift in the presence of 25 pg/mL IL-6 (Figure 6), while the BSA passivated (8,7) chirality also demonstrated a significant red-shift.

**Figure 7:**
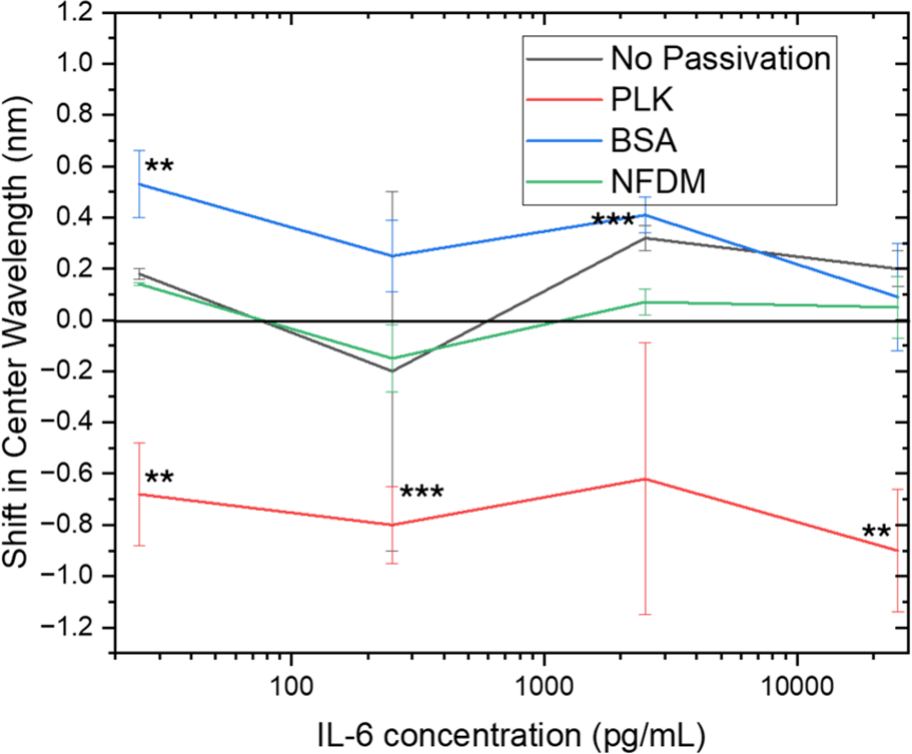
Comparison of response for three passivation agents to IL-6 concentration increases in human serum. For the (8,7) chirality, the PLK passivated nanosensor responds to the presence of all 4 concentrations of IL-6 with a blue-shifted center wavelength. The BSA passivated nanosensor responds to presence of 25, 250, and 2500 pg/mL concentrations with a red-shifted center wavelength. *p *<* 0.05; **p *<* 0.01; ***p *<* 0.001.

Upon examination of all 4 concentrations evaluated, we found that the PLK passivated (8,7) chirality demonstrated a substantial shift across all four concentrations (>0.6 nm) (Figure 7). Notably, both (9,4) and (7,6) (Figure S29A) chiralities demonstrated significant shifts in response to IL-6 after PLK passivation. In all cases, the shift in the center wavelength was calculated relative to a passivated control in human serum with no IL-6 spiked in. We believe that it is likely that certain chiralities demonstrate superior detection at low concentrations due to the variability in ssDNA-SWCNT interactions across each of the chiralities which vary in diameter and helical pitch. Next, we studied the stability of the signal generated by passivated nanosensor. We also found that the PLK passivated sensor response is stable for at least 3 h after passivation (Figure S30). In addition to PLK passivation, BSA passivated (9,4) nanosensor chirality demonstrated a significant shift in response to 250 pg/mL IL-6 in human serum (Figure 7).

We then analyzed and compared the performance of nonpassivated nanosensor with passivated nanosensors across all four chiralities to assess the utility of passivation as a strategy. We found that the PLK passivated nanosensor provides significant improvement in detection of IL-6 over nonpassivated nanosensor (Figures S31–S35). (7,5) and (8,7) chiralities demonstrated significant shifts in the presence of 25 pg/mL IL-6 (Figures S32, S33) and the (7,6) demonstrated a significant shift in the presence of 250 pg/mL (Figure S34). All four chiralities that we observed exhibited significant shifts in the presence of 2,500 pg/mL (Figures S32–S35) while the (8,7) and (9,4) demonstrated significant shifts in the presence of 25,000 pg/mL (Figures S33, S35). Of the other passivation agents investigated (BSA and NFDM), only the BSA-passivated nanosensor demonstrated any statistically significant performance improvement over the nonpassivated nanosensor, specifically the (9,4) chirality with 250 pg/mL IL-6 (Figure S35). Overall, these findings demonstrate that a multichiral nanosensor with highly sensitive and quantitative IL-6 detection in human serum gives rise to the possibility of rapid detection at home or at the patient’s bedside.

Based on a substantial literature search, this work represents a dramatic increase in the sensitivity of SWCNT-based optical sensors in human serum and the first such for IL-6 inflammatory cytokines. In prior studies, antibody-conjugated SWCNT-based optical nanosensors for cancer biomarkers have shown a limit of detection (LOD) of 33,800 pg/mL (2.6 nM) for the ovarian cancer biomarker HE-4 in human serum.^3^ Here, we demonstrated that the PLK passivated nanosensor detects IL-6 concentrations as low as 25 pg/mL in human serum, 1,352 times lower than that which was previously reported. It also provides detection across 4 orders of magnitude, from 25 pg/mL to 25,000 pg/mL.^3^ IL-6 levels in serum are reported to be above 20 pg/mL in patients diagnosed with cancer, neurological diseases, sepsis, and COVID-19.^28^ Concentration levels above 500 pg/mL and 7,500 pg/mL correlate with patient mortality in 11% and 37% of sepsis cases, respectively.^65^ The IL-6 nanosensor presented here demonstrates detection, in clinically relevant serum conditions, well within these diagnostic concentration ranges. In addition to the clearly established bedside diagnostic potential of this nanosensor construct, combining this with the established in vivo detection applicability of SWCNTs^26,30^ portends a strong potential for implantable diagnostic device design.

**Figure 8:**
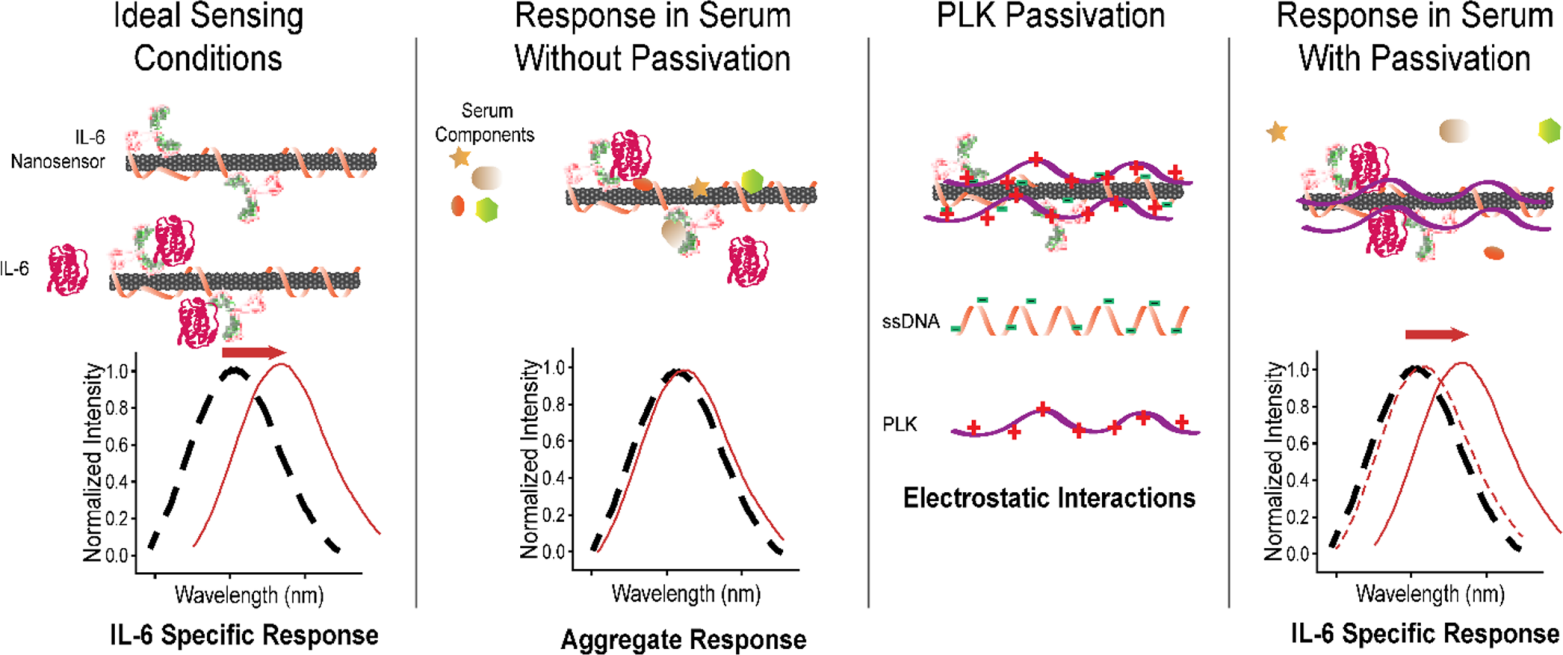
Diagram of the proposed mechanism of action for PLK-induced sensing of IL-6 in human serum.

We also found that the ability to detect different IL-6 concentrations differs across nanotube chiralities for a given passivation agent. Many factors influence the success of passivation—size, conformation, hydrophobicity, and ionic charge of a given passivation agent, as well as the atomic composition, surface roughness, and curvature of the nanomaterial being passivated, plus the pH, temperature, and ionic strength of the local solute environment.^66^ It is known that SWCNT chiralities differ in surface composition and size due to varying ssDNA wrapping abilities.^67−70^ In this study, it is likely that the charge–charge interactions of PLK with ssDNA dominated the passivation process (Figure 8).^55^ Different ssDNA sequences are known to provide varying degrees of surface coverage for specific SWCNT chiralities, which could potentially affect the extent of PLK passivation success across different chiralities.^7,68−70^ We propose that these variations contribute to variations in functionality for a given SWCNT chirality.

## Conclusions

In this work, we observed the highly sensitive detection of the clinically relevant cytokine IL-6 in human serum across a broad functional range of concentrations. To achieve this, we first screened three classes of biomolecules—proteins, polymers, and amphiphiles—as potential passivating agents. We found that BSA, NFDM, and casein—all proteins—and PLK, a polymer, were the most successful passivation agents in FBS screening. We found that 50× and 100× mass ratios of passivating agents to SWCNTs were more successful than 5× and 25× mass ratios. These results were corroborated by absorption spectroscopy, demonstrating stable surface coverage for each. With PLK and/or BSA passivation, an engineered IL-6 nanosensor demonstrated clinically relevant detection as low as 25 pg/mL, ranging up to 25,000 pg/mL upon observation of multiple nanotube chiralities. We expect this study to provide rational strategies to screen interference from heterogeneously formed coronas upon introduction to complex sensing conditions, improving the selectivity and sensitivity of, specifically, SWCNT-based optical nanosensors and, more broadly, other nanosensors, for clinical applications.

## Supporting Information Available

Chemical structures of polymers and surfactants used in screens, response of (7,5) and (9,5) chiralities, NIR fluorescent spectra, spectral shifts over time, image of SWCNT sensor in solution, and IL-6 nanosensor characterization and response in buffer and human serum (PDF)
