# Supplementary material for: Optical Nanosensor Passivation Enables Highly Sensitive Detection of the Inflammatory Cytokine Interleukin-6
Source: ACS Appl Mater Interfaces. 2024 May 15;16(21):27102–13. doi: 10.1021/acsami.4c02711 (PMC11145596; doi:10.1021/acsami.4c02711)
Supplement: Supplementary file 1 — am4c02711_si_001.pdf [file am4c02711_si_001.pdf]

Supporting Information:

Optical Nanosensor Passivation Enables Highly Sensitive Detection of the Inflammatory Cytokine Interleukin-6

Pooja Gaikwad[1,2], Nazifa Rahman[1], Rooshi Parikh[1], Jalen Crespo[1], Zachary Cohen[1], and Ryan M. Williams[1,2]*

[1]Department of Biomedical Engineering, The City College of New York, New York, NY 10031, United States of America

[2]PhD Program in Chemistry, The Graduate Center of The City University of New York, New York, NY 10016, United States of America

*correspondence to rwilliams4@ccny.cuny.edu

Figures

Class	Structure
Polymers	• Polyethylene Glycol<br>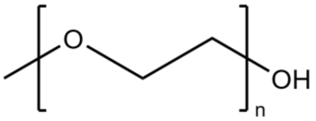
	• Polyethylene Imine<br>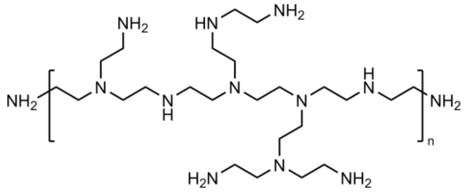
	• Poly-L-Lysine<br>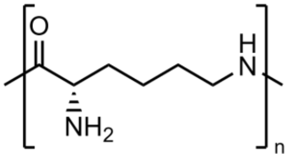
Surfactants	• 16:0 PE2000PEG<br>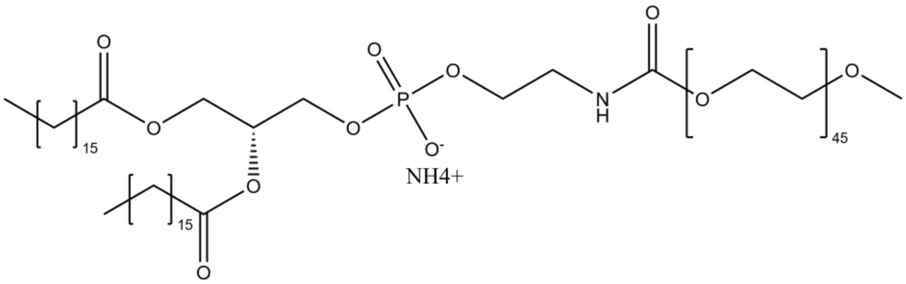
	• DSPE-PEG-amine<br>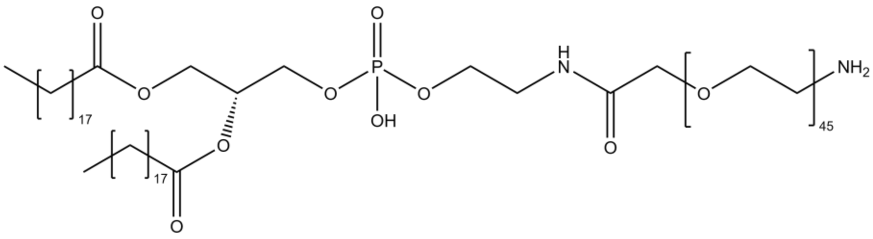

Figure S1. Chemical structures of passivation agents.

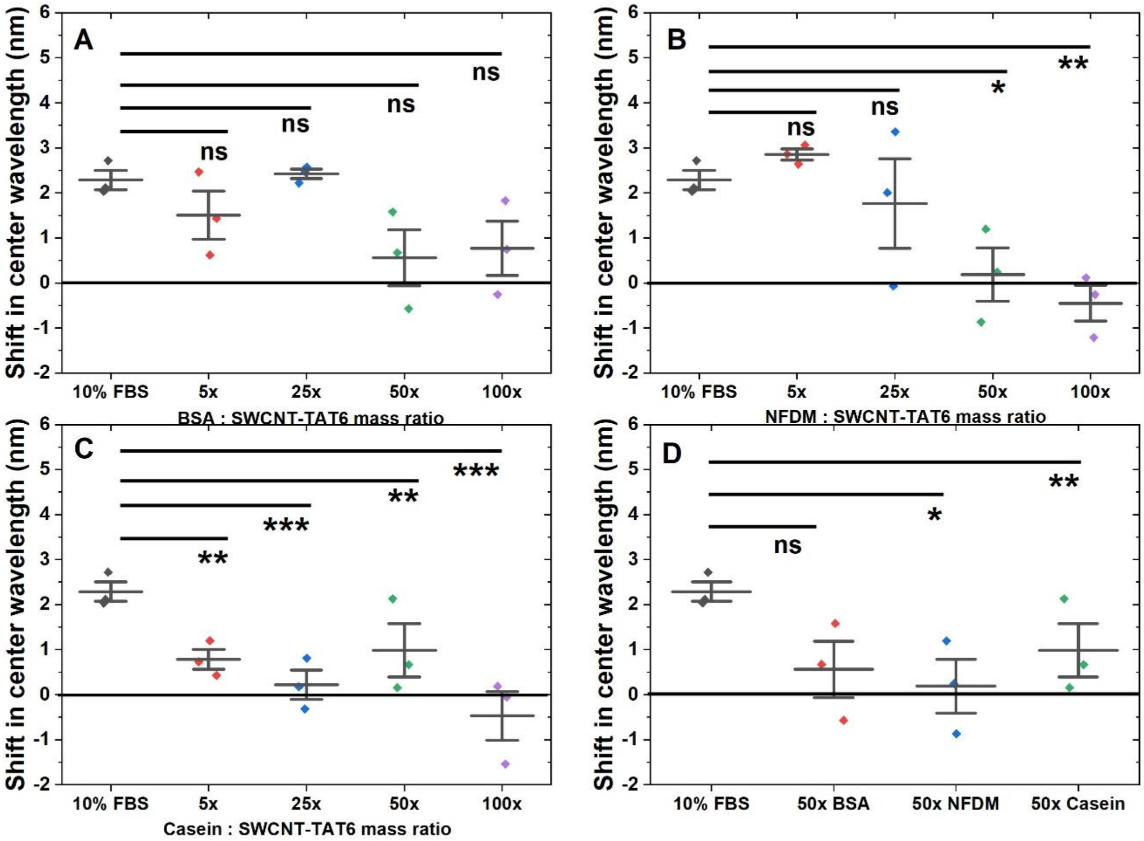

Figure S2. Change in (7,5) fluorescence peak upon challenging protein passivations with FBS. Successful screening of serum interference was exhibited (A) by none of the mass ratios of BSA passivation, n=3(B) by only 50x and 100x mass ratio for NFDM passivation, n=3, (C) by all mass ratios for casein passivation, n=3, (D) The 50x mass ratio is the lowest common passivation ratio shown to be successful amongst protein passivation, n=3,

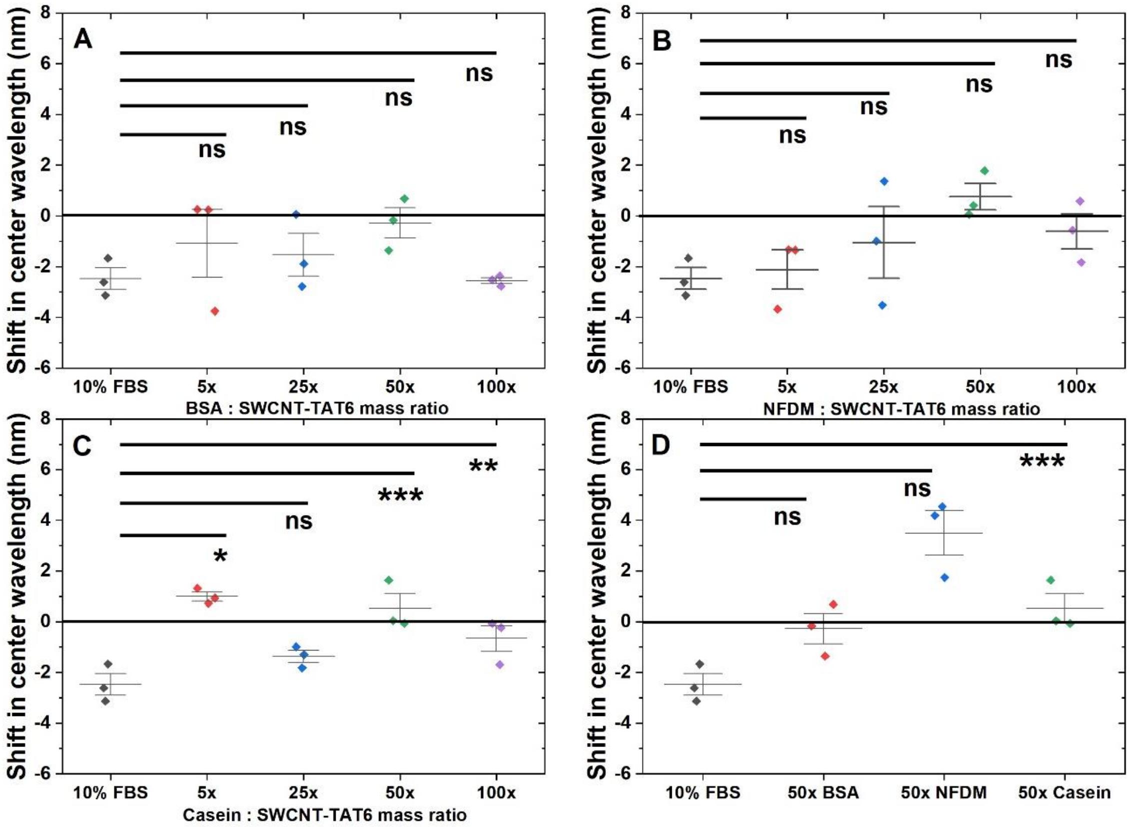

Figure S3. Change in (9,5) fluorescence peak upon challenging protein passivations with FBS. Successful screening of serum interference was exhibited by none of the mass ratios for (A) BSA passivation, n=3 and (B) for NFDM passivation, n=3. (C) by all except 25x mass ratios for casein passivation, (D) by NFDM and casein, for only 50x mass ratio.

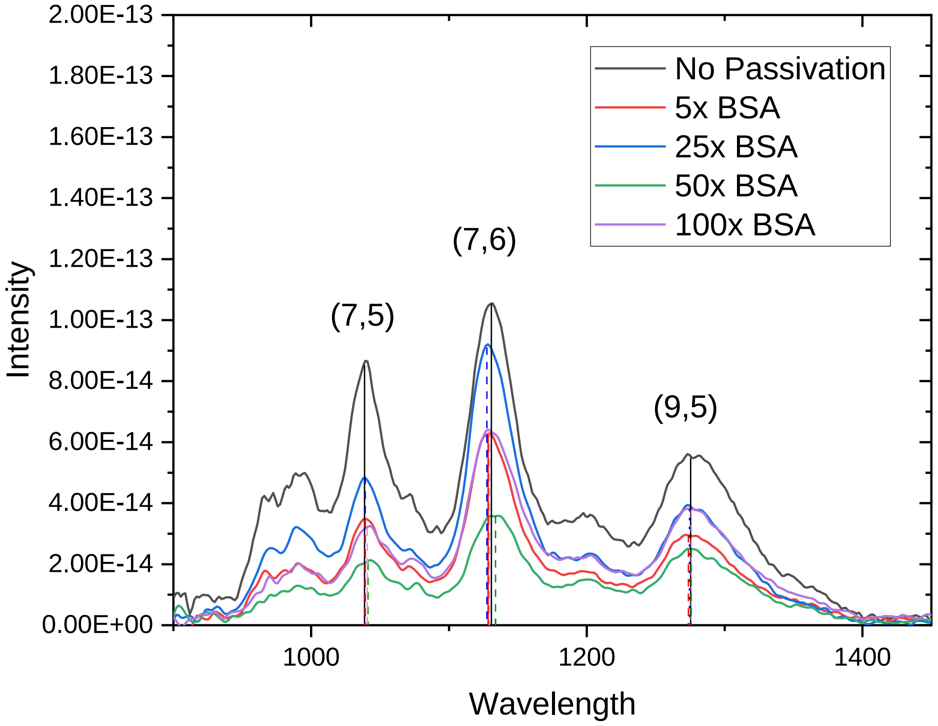

Figure S4. Non-passivated vs BSA passivation spectra. For all three chiralities evaluated--(7,5), (7,6), and (9,5)--for all BSA : SWCNT-$(TAT)_6$ mass ratio passivations, center wavelength of SWCNT-$(TAT)_6$ decreased in intensity upon passivation. The highest decrease in the intensity was observed upon 50 x BSA passivation. For the (7,6) chirality, all passivations except for BSA resulted in center wavelength blue shift of SWCNT-$(TAT)_6$. For the (7,5) chirality, all mass ratios showed a red shift with 50x BSA passivation, causing a substantial shift. For the (9,5) chirality, all mass ratios showed a blue shift.

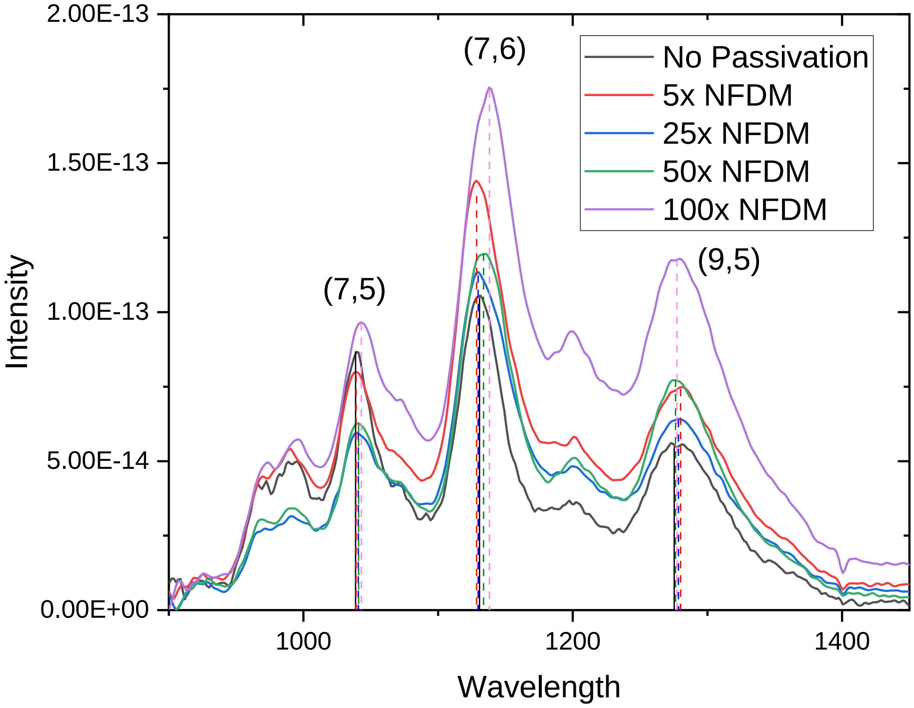

Figure S5. Non-passivated vs NFDM passivation spectra. For all three chiralities evaluated--(7,5), (7,6), and (9,5)—with NFDM passivation.

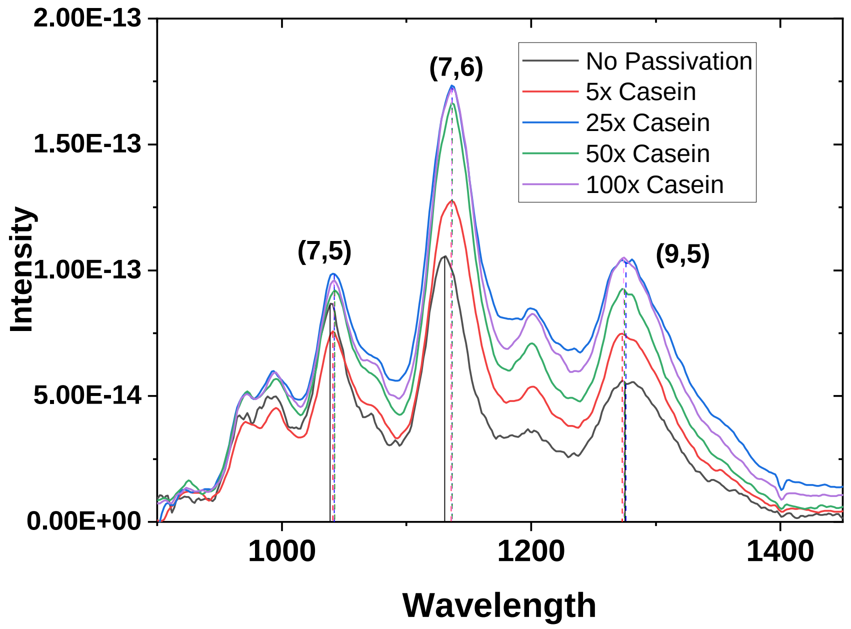

Figure S6. Non-passivated vs casein passivated SWCNT spectra. For all three chiralities evaluated--(7,5), (7,6), and (9,5)—with casein passivation.

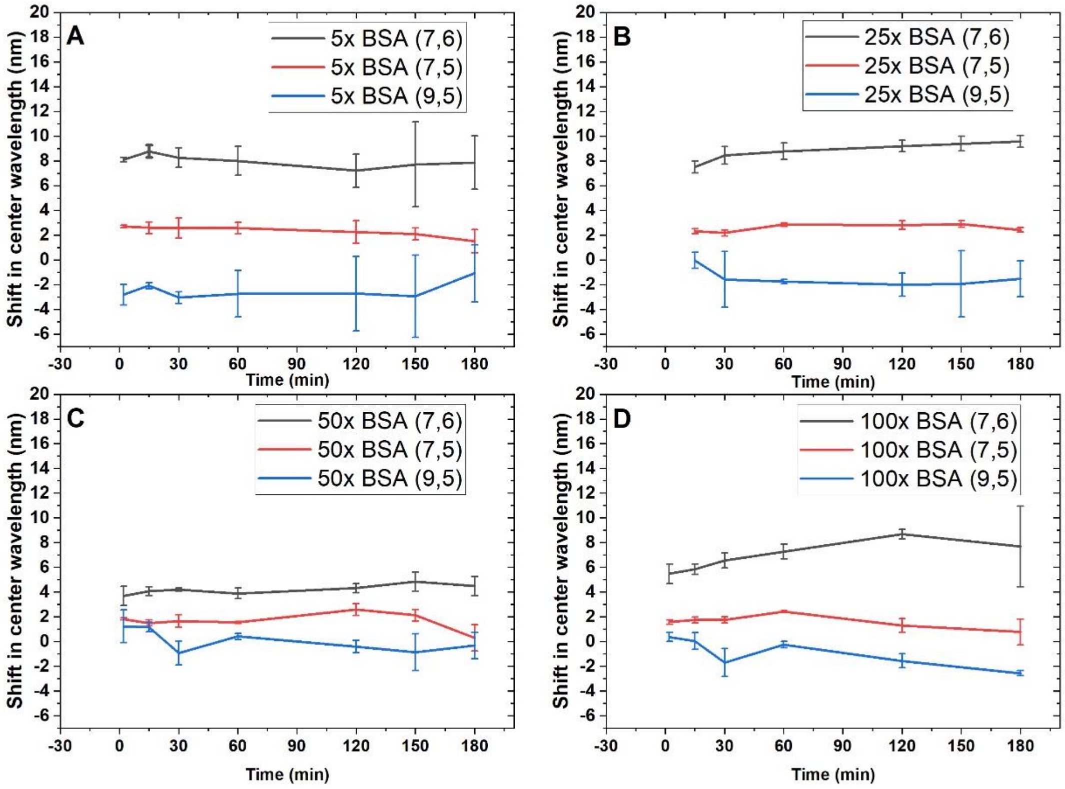

Figure S7. Change in all fluorescence peaks over time after addition of FBS to BSA-passivated SWCNT. (A) For 5x BSA passivation ratio, (B) For 25x BSA passivation ratio, (C) For 50x BSA passivation ratio, (D) For 100x BSA passivation ratio.

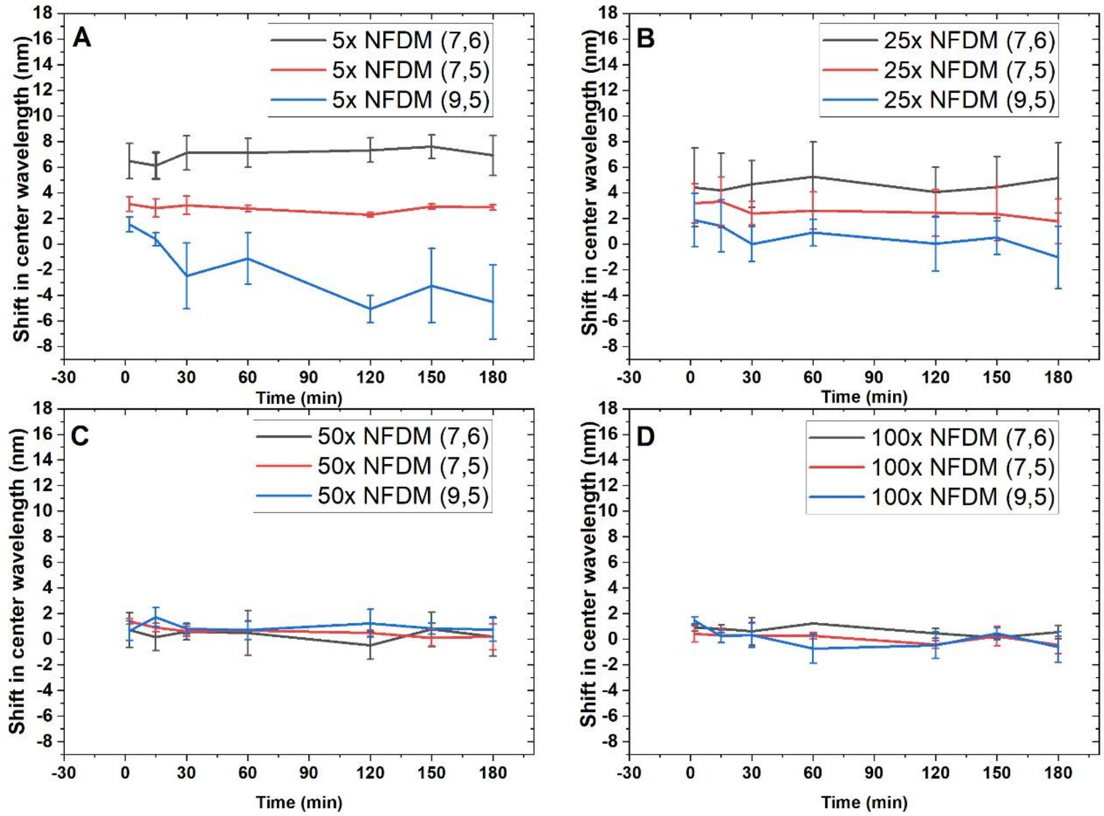

Figure S8. Change in all fluorescence peaks over time after addition of FBS to NFDM-passivated SWCNT. (A) For 5x NFDM passivation ratio, (B) For 25x NFDM passivation ratio, (C) For 50x NFDM passivation ratio, (D) For 100x NFDM passivation ratio.

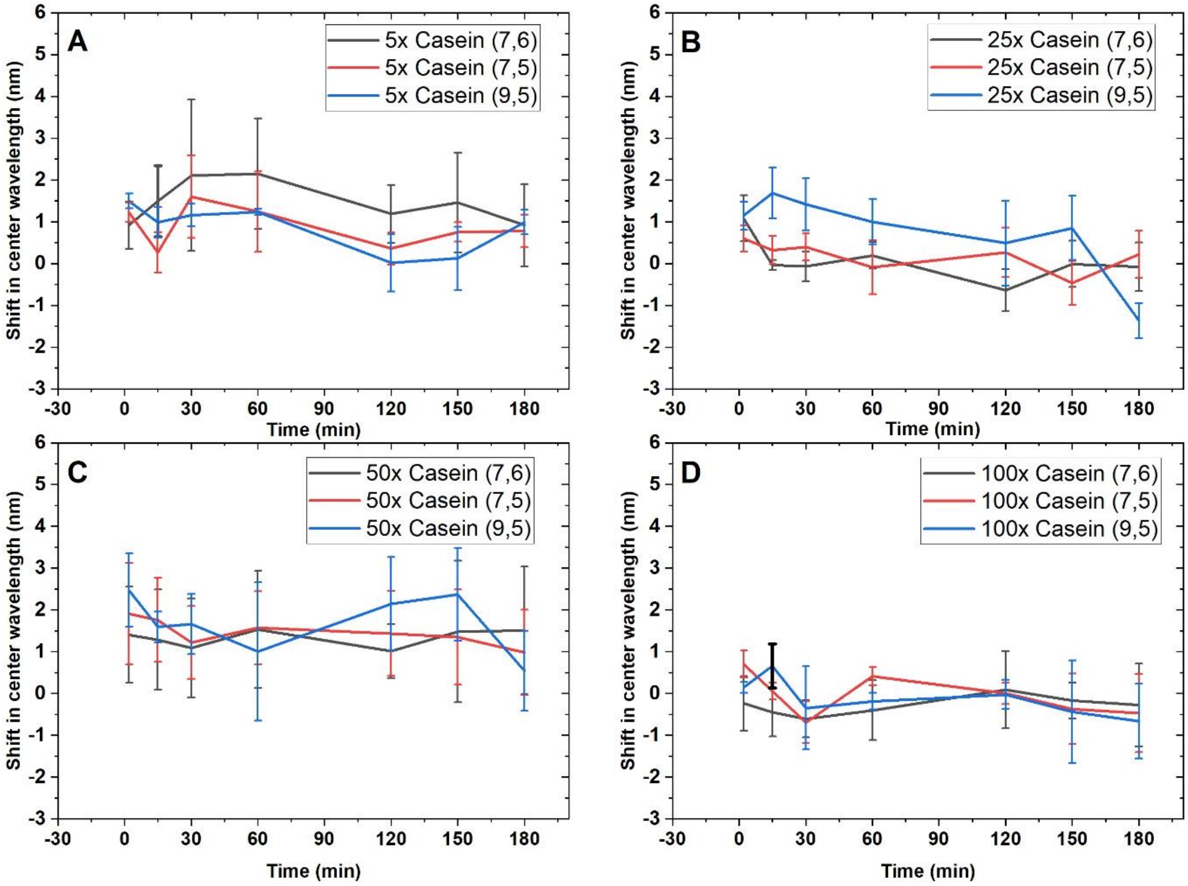

Figure S9. Change in all fluorescence peaks over time after addition of FBS to casein-passivated SWCNT. (A) For 5x casein passivation ratio, (B) For 25x casein passivation ratio, (C) For 50x casein passivation ratio, (D) For 100x casein passivation ratio.

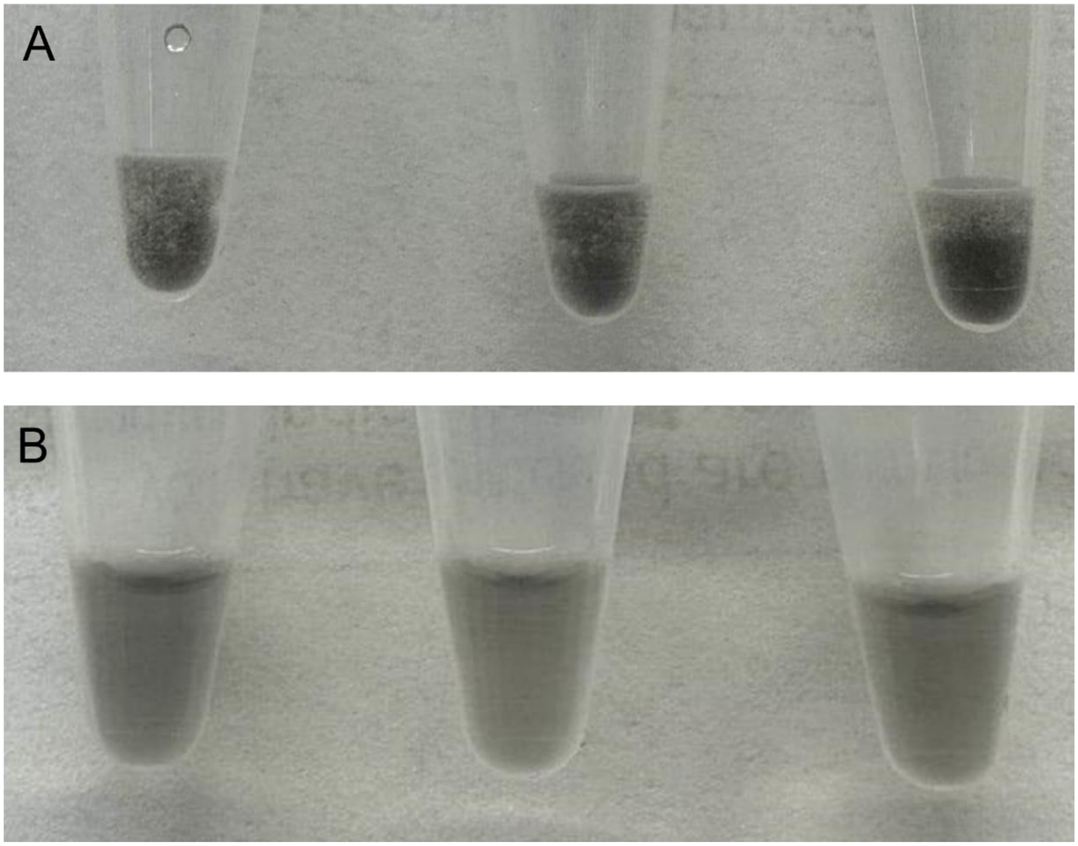

Figure S10. Images of SWCNT flocculation after PEI passivation. (A) 12 hours after passivation with 50x PEI, aggregation of 10 mg/L SWCNT-$(TAT)_6$ was observed. (B) Stable solution of 50x BSA passivated 10 mg/L SWCNT-$(TAT)_6$ for at least 48 hours.

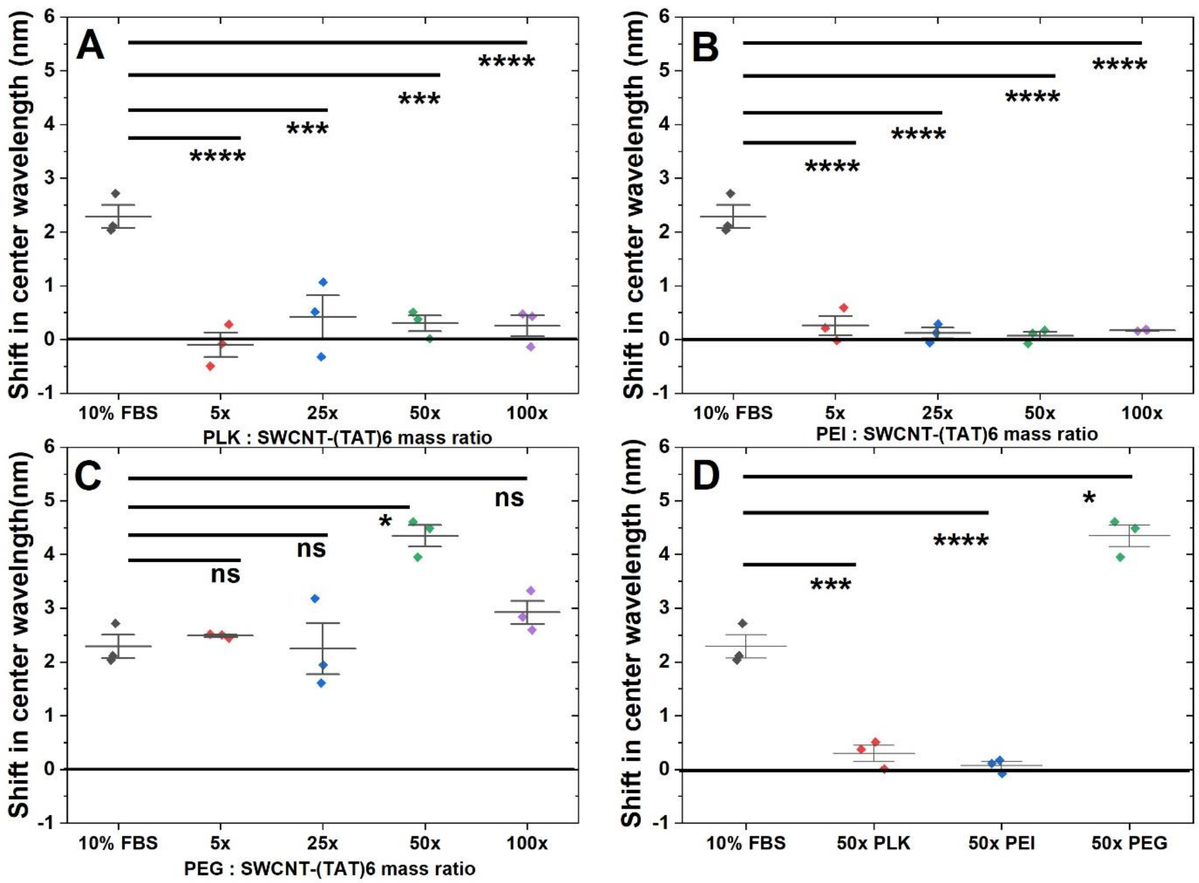

Figure S11. Change in (7,5) fluorescence peak upon challenging polymer passivation with FBS. Successful screening of serum interference was exhibited by all mass ratios of (A) PLK passivations, n=3 and (B) PEI passivations, n=3. No mass ratios of (C) PEG passivations demonstrated successful screening, n=3, (D) 50x mass ratio shows screening effect for PLK as well as PEI passivation.

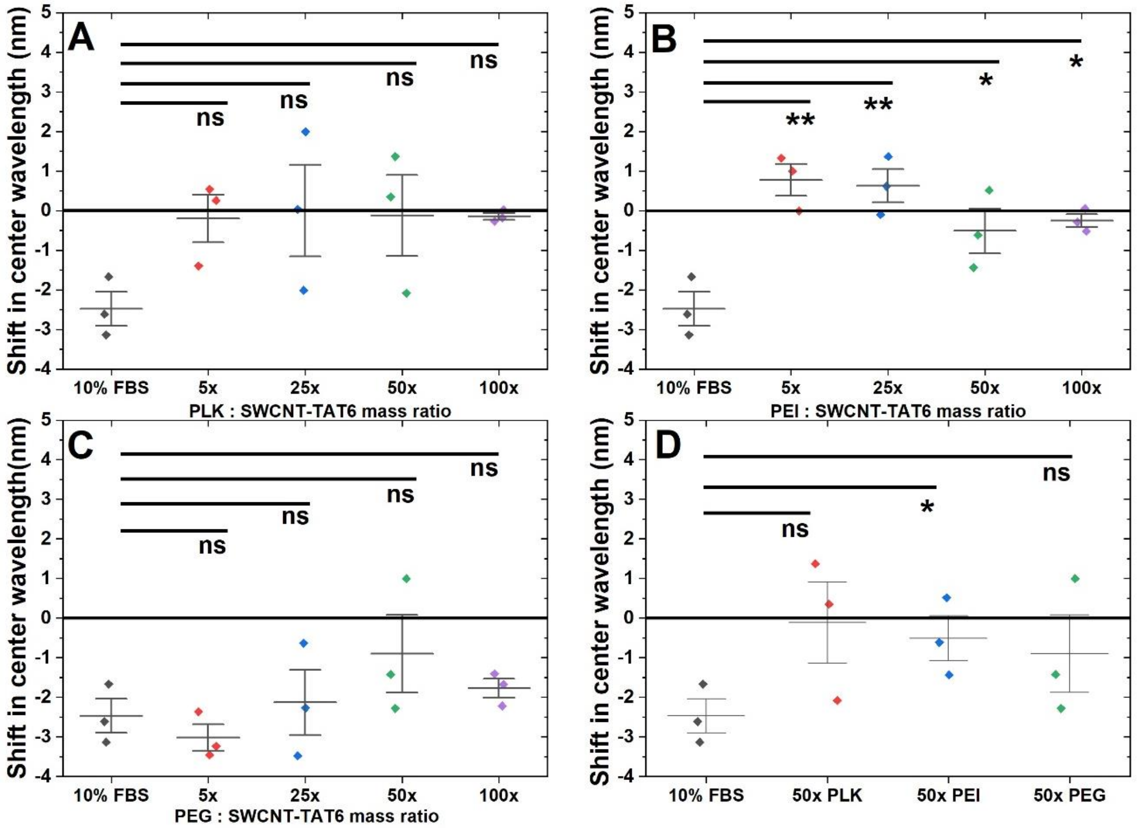

Figure S12. Change in (9,5) fluorescence peak upon challenging polymer passivation with FBS. Successful screening of serum interference was exhibited by none of the mass ratios (A) for PLK passivations, n=3, all of the mass ratios (B) for PEI passivations, n=3, mean ± SD, and none of the mass ratios (C) for PEG passivations, n=3, mean ± SD. (D) For 50x mass ratio, none of the polymer agents were successful.

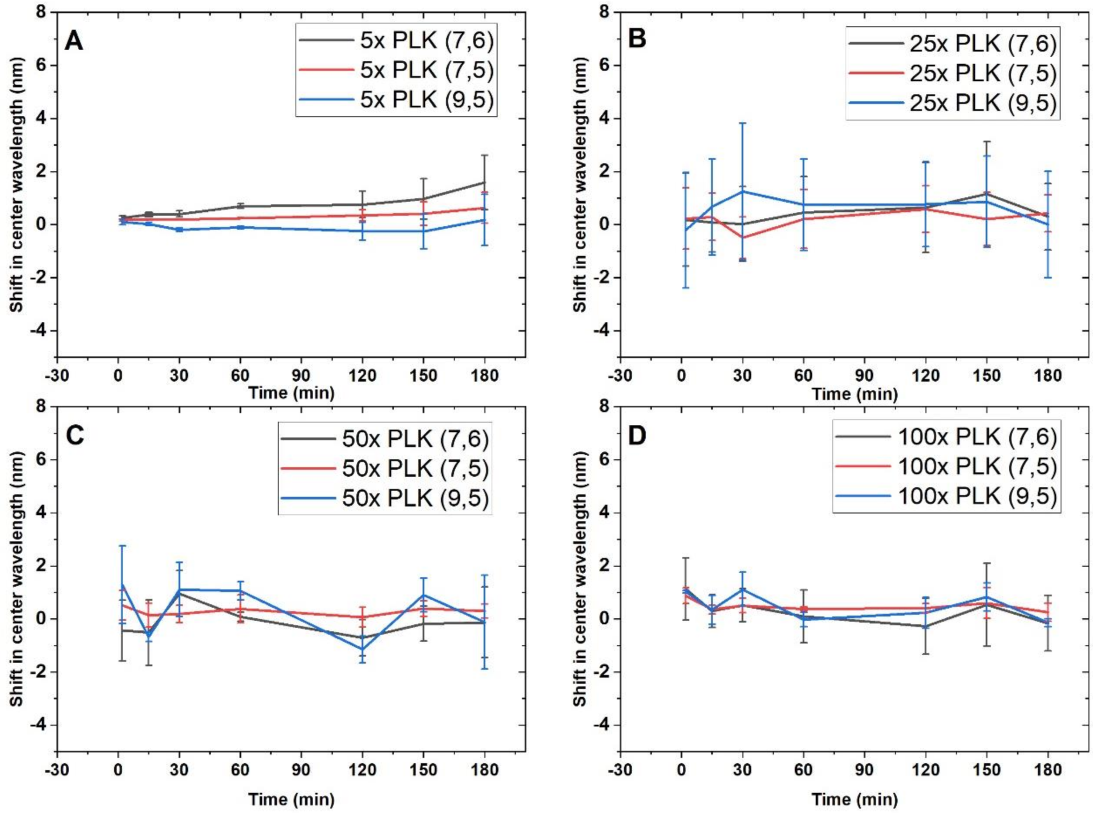

Figure S13. Change in all fluorescence peaks over time after passivation after FBS addition for PLK-passivated SWCNT (A) For 5x PLK passivation ratio, (B) For 25x PLK passivation ratio, (C) For 50x PLK passivation ratio, (D) For 100x PLK passivation ratio.

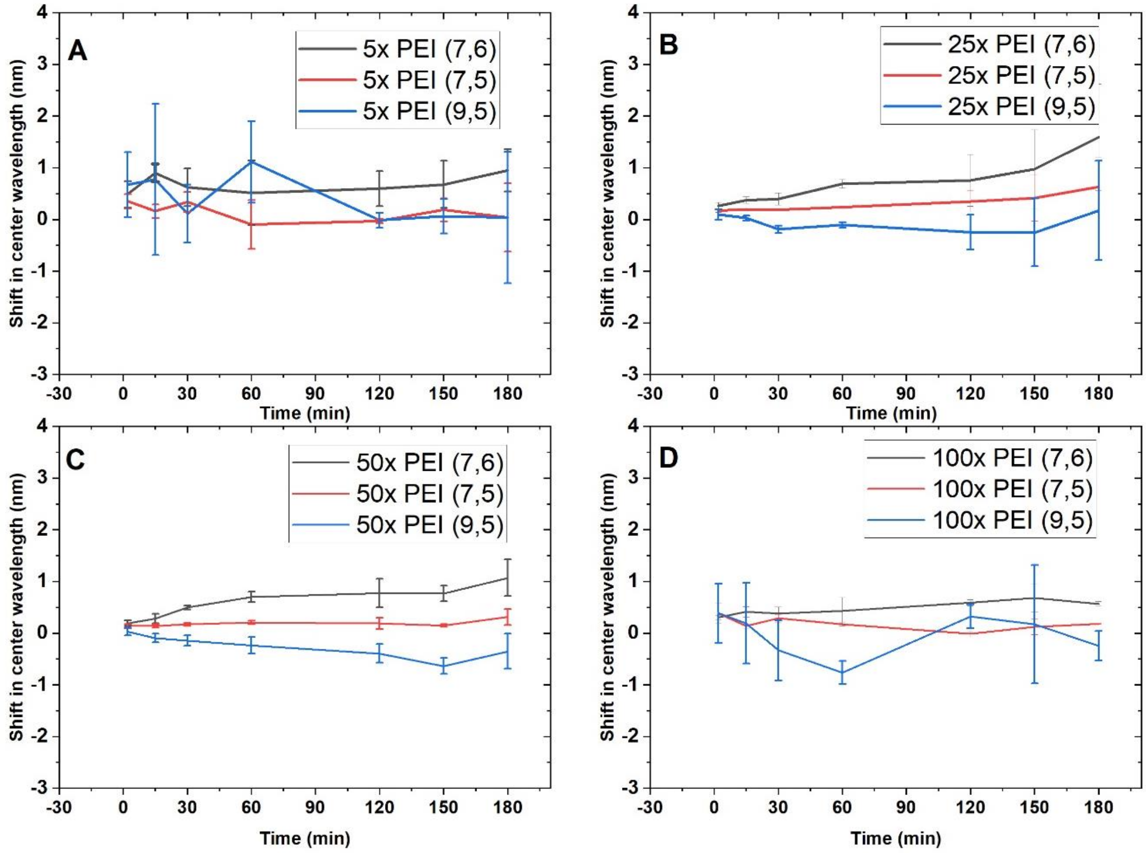

Figure S14. Change in all fluorescence peaks over time after passivation after FBS addition for PEI-passivated SWCNT. (A) For 5x PEI passivation ratio, (B) For 25x PEI passivation ratio, (C) For 50x PEI passivation ratio, (D) For 100x PEI passivation ratio

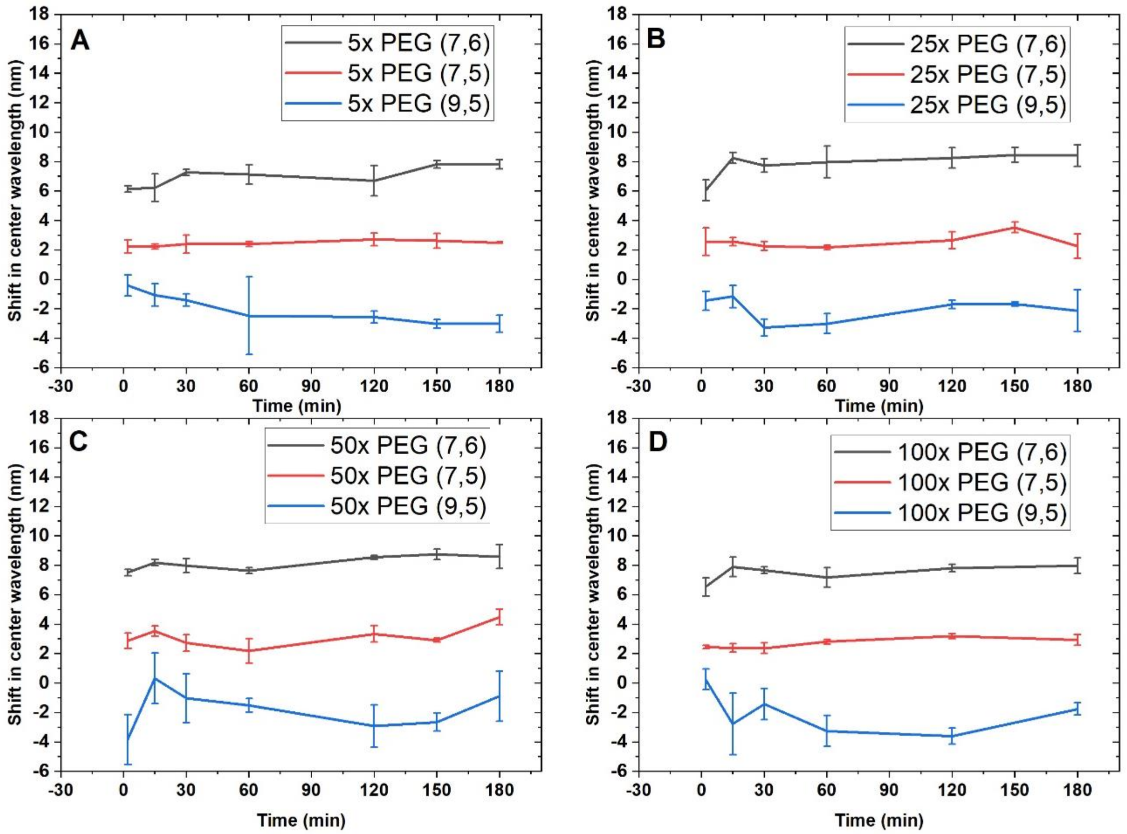

Figure S15. Change in all fluorescence peaks over time after addition of FBS for PEG-passivated SWCNT. (A) For 5x PEG passivation ratio, (B) For 25x PEG passivation ratio, (C) For 50x PEG passivation ratio, (D) For 100x PEG passivation ratio.

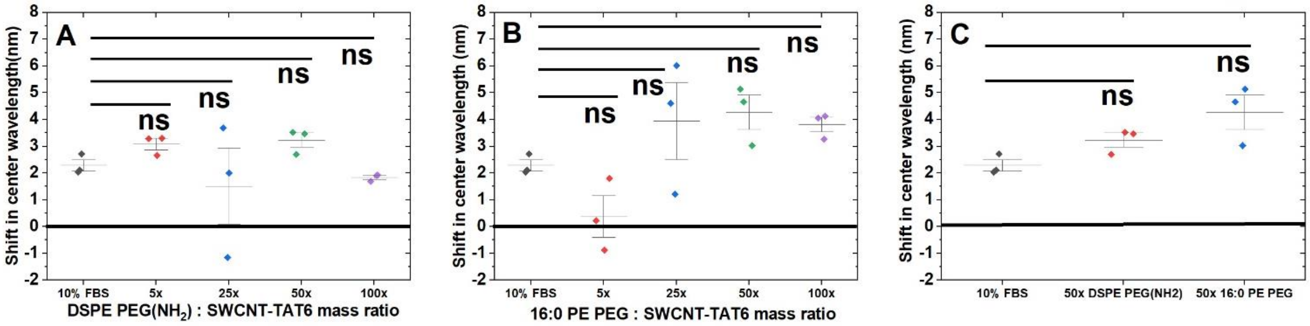

Figure S16. Change in (7,5) fluorescence peak upon challenging phospholipid passivation with FBS. Successful screening of serum interference was exhibited by none of the mass ratios (A) For DSPE PEG (NH_2) passivations, n=3, And (B) For 16:0 PE PEG passivations, n=3, mean ± SD. 10% FBS (2.3 ± 0.4 nm), 5x 16:0 PE PEG (0.4 ± 1.3 nm), 25x 16:0 PE PEG (3.9 ± 2.5 nm), 50x 16:0 PE PEG (4.3 ± 1.1 nm), 100x 16:0 PE PEG (3.8 ± 0.5 nm). FBS and 5x 16:0 PE PEG (2.6 nm, p=0.2), FBS and 25x 16:0 PE PEG (0.98 nm, p=0.8), FBS and 50x 16:0 PE PEG (1.3 nm, p=0.7), FBS and 100x 16:0 PE PEG (0.9 nm, p=0.9). (C) For 50x mass ratio, both phospholipid passivations do not screen serum interference.

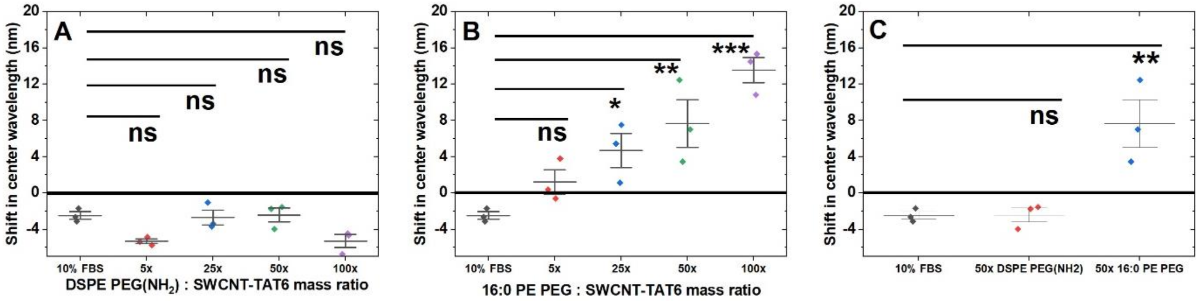

Figure S17 Change in (9,5) fluorescence peak upon challenging phospholipid passivation with FBS. Successful screening of serum interference was exhibited by (A) none of the mass ratios For DSPE PEG (NH_2) passivations, n=3, and (B) For 16:0 PE PEG passivations, n=3, (C) For 50x mass ratio, both the phospholipid passivations do not screen serum interference.

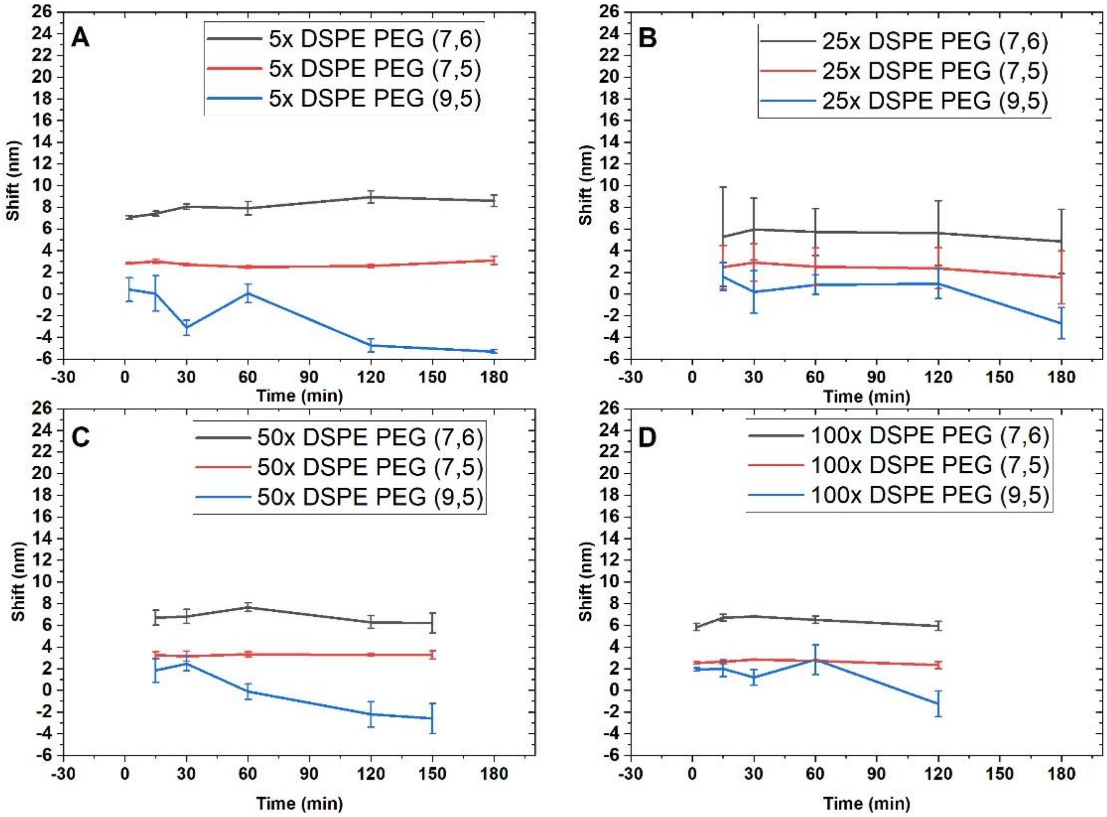

Figure S18. Change in all fluorescence peaks over time after addition of FBS to DSPE-PEG (NH_2)-passivated SWCNT. (A) For 5x DSPE PEG (NH_2) passivation ratio, (B) For 25x DSPE PEG (NH_2) passivation ratio, (C) For DSPE PEG (NH_2) 50x passivation ratio, (D) For DSPE PEG (NH_2) 100x passivation ratio.

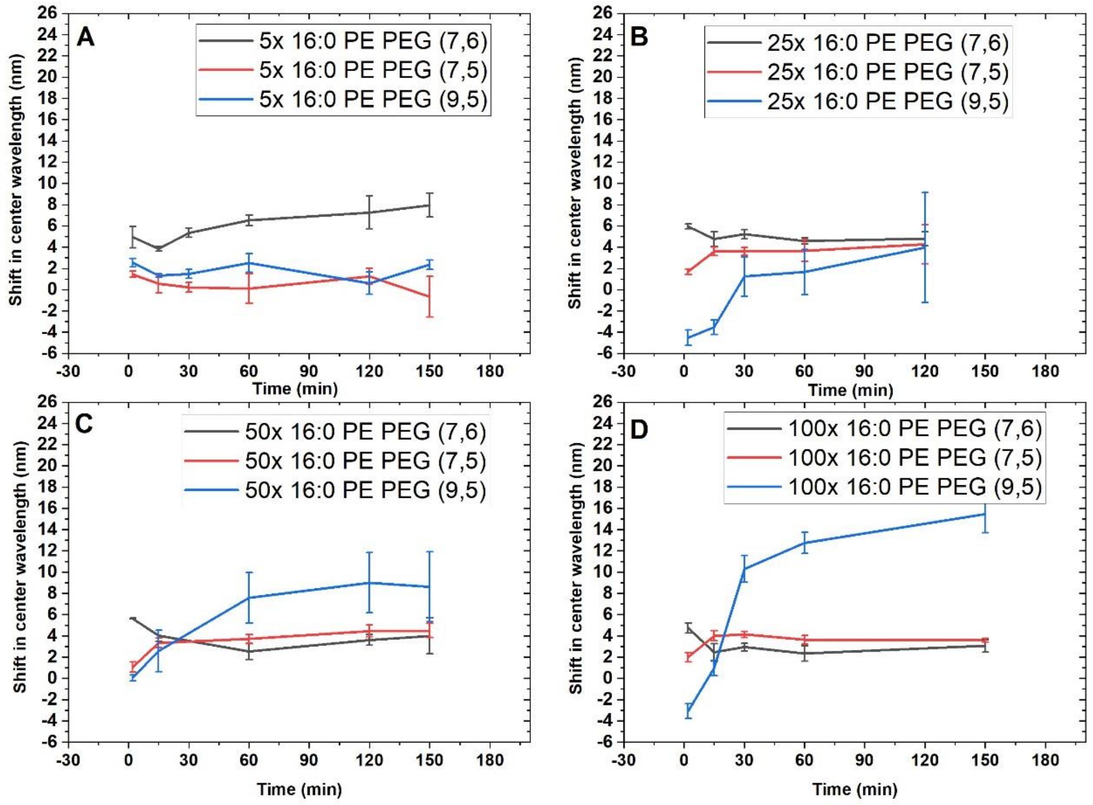

Figure S19. Change in all fluorescence peaks over time after FBS addition to PE2000PEG-passivated SWCNT. (A) For 5x PE2000PEG passivation ratio. (B) For 25x DSPE PE2000PEG passivation ratio, (C) For 50x PE2000PEG passivation ratio, and (D) For 100x DSPE PEG PE2000PEG passivation ratio.

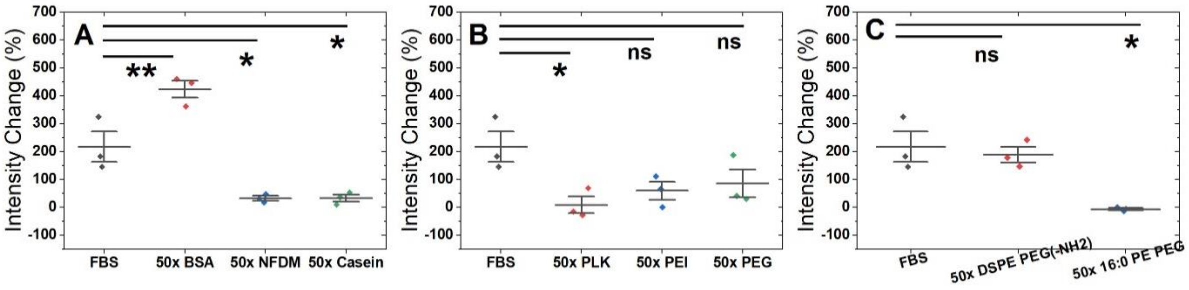

Figure S20. Change in (7,6) fluorescence peak intensity of SWCNT-(TAT)$_6$ in the presence of 50x mass ratio passivation agents compared to FBS alone. (A) Only NFDM and casein of the proteins passivations screened effect of FBS intereference on fluorescence intensity of

SWCNT-(TAT)6. (B) Polymers and (C) phospholipids were screened as well, and only poly-L-lysine and 16:0 PE PEG passivation passivations were successful.

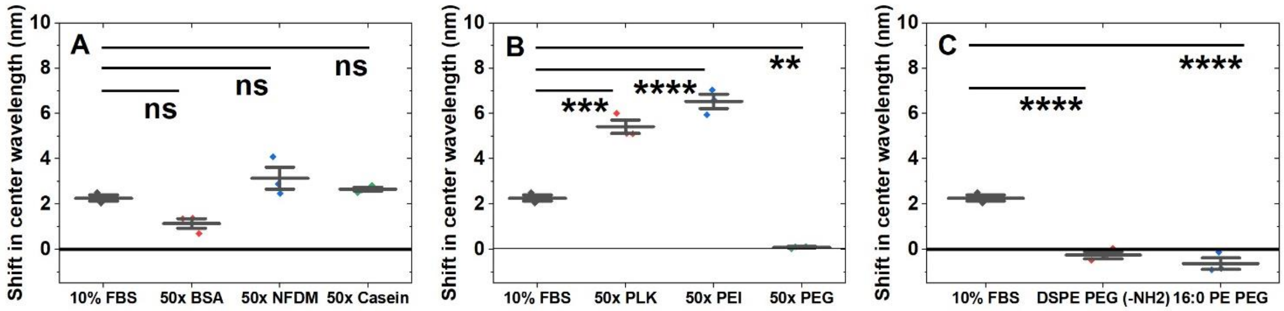

Figure S21. Change in 1037 nm absorbance peak of SWCNT-(TAT)6 in presence of 50x mass ratio passivation agents compared to FBS alone. (A) Protein passivation agents, (B) polymer passivation agents, and (C) phospholipid passivation agents were evaluated for their ability to prevent center wavelength shift for this center wavelength.

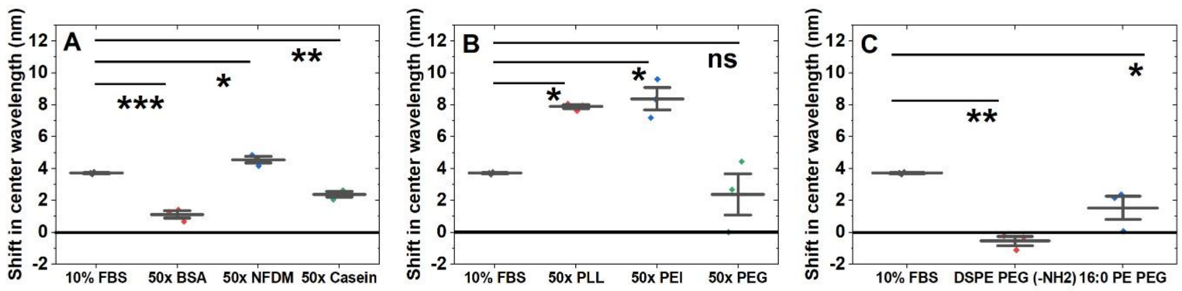

Figure S22. Change in 1270 nm absorbance peak of SWCNT-(TAT)6 in the presence of 50x mass ratio passivation agents compared to FBS. (A) Protein passivation agents, (B) polymer passivation agents, and (C) phospholipid passivation agents were evaluated for their ability to prevent center wavelength shift for this center wavelength. Only NFDM, PLK, and PEI show shift higher than serum. 1270 nm absorbance peak includes (9,5) chirality absorbance.

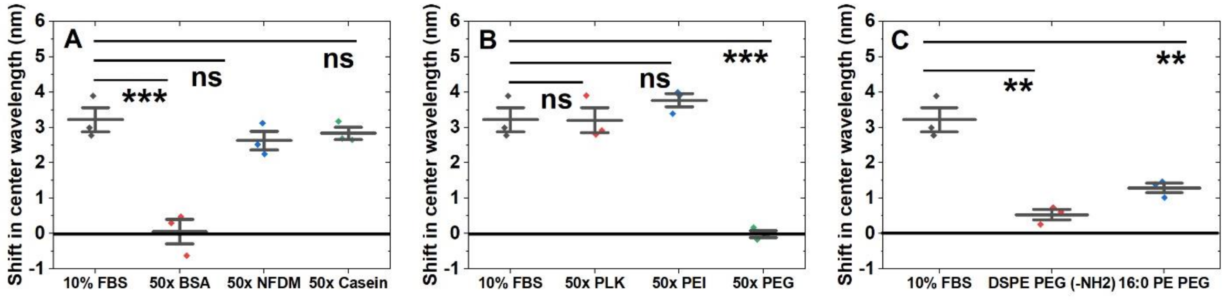

Figure S23. Change in 990 nm absorbance peak of SWCNT-(TAT)6 in the presence of passivation agents compared to FBS. (A) For protein passivations, n=3, mean ± SD. (10% FBS = 3.2 ± 0.6 nm), 50x BSA (0.05 ± 0.6 nm), 50x NFDM (2.6 ± 0.4 nm), 50x casein (2.8 ± 0.3 nm). FBS and 50x BSA (3.2 nm, p=9.5E-4), FBS and 50x NFDM (0.6 nm, p=0.46), FBS and

50x casein (0.38 nm, p=0.7). (B) For polymer passivations, n=3, mean ± SD. (10% FBS = 3.22 ± 0.6 nm), 50x PLK (3.2 ± 0.6 nm), 50x PEI (5.2 ± 1 nm), 50x PEG (-1.2 ± 0.2 nm). FBS and 50x PLK (1.5E-2 nm, p=1), FBS and 50x PEI (0.55 nm, p=0.44), FBS and 50x PEG (3.2 nm, p=4.4E-4). (C) For phospholipid passivations, n=3, mean ± SD. (10% FBS = 3.2 ± 0.6 nm), 50x DSPE PEG (NH_2) (5.4 ± 0.3 nm) and 50x 16:0 PE PEG (1.3 ± 0.2 nm). FBS and 50x DSPE PEG (NH_2) (2.7 nm, p=3E-3), and FBS and 50x 16:0 PE PEG (1.9 nm, p=1E-2).

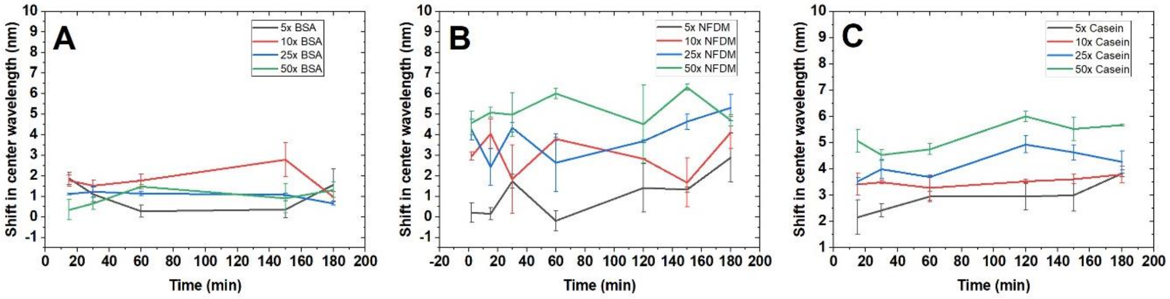

Figure S24. Change in center wavelength of 1130 nm absorption peak over time after addition of protein passivation agents. (A) For all BSA mass ratio passivation, (B) For all Non-fat dry milk mass ratio passivations, and (C) For all casein mass ratio passivations.

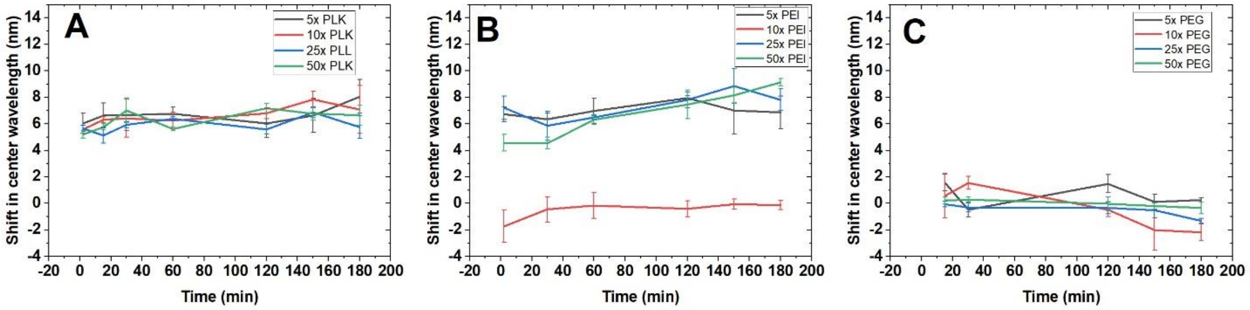

Figure S25. Change in center wavelength of 1130 nm absorption peak over time after addition of polymer passivation agents. (A) For all poly-L-lysine mass ratio passivations, (B) For all polyethylene imine mass ratio passivations, and (C) For all polyethylene glycol mass ratio passivations.

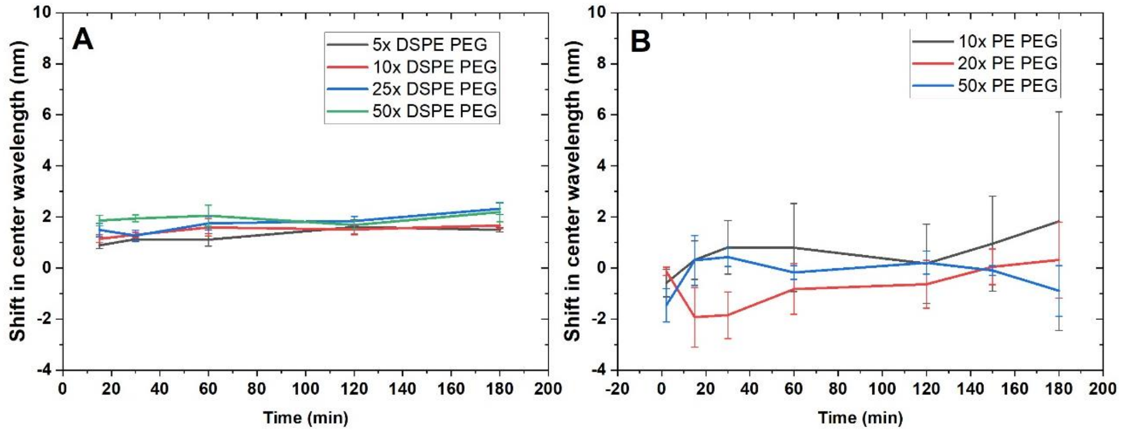

Figure S26. Change in center wavelength of 1130 nm absorption peak over time after addition of phospholipid passivation agents. (A) For all DSPE PEG (NH_2) mass ratio passivations and (B) For all 16:0 PE 2000 PEG mass ratio passivations.

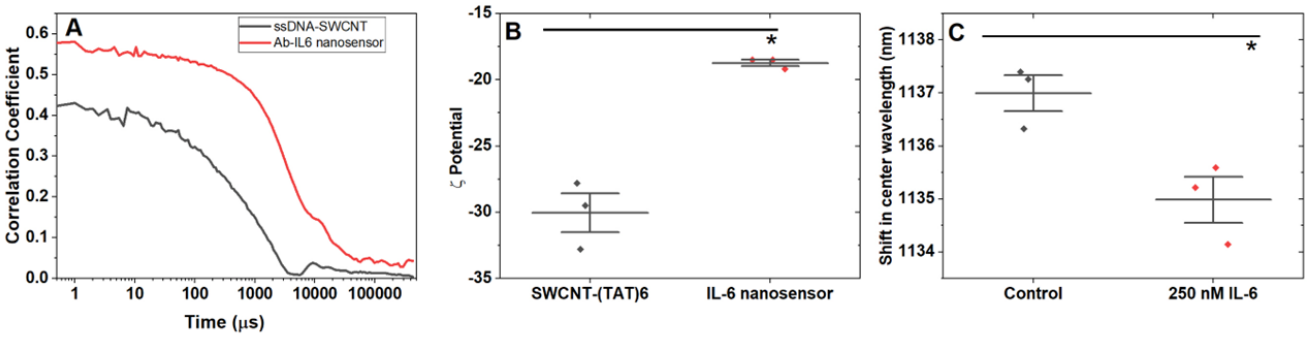

Figure S27. Characterization and in vitro performance of an engineered IL-6 nanosensor. Successful conjugation of the antibody-based IL-6 nanosensor was assessed by (A) comparison of decay in correlation coefficient as function of time for SWCNT-$(TAT)_6$ and IL-6 Antibody (Ab) conjugated SWCNT-$(TAT)_6$, (B) Change in zeta potential for SWCNT-$(TAT)_6$ (-30 ± 2.5 mV) compared to the IL-6 nanosensor (-18.7 ± 0.4 mV) (difference in means = -11.3 mV, p=0.01, two-tailed t-test). (C) Performance of the IL-6 nanosensor was assessed by comparing change in the emission peak for IL-6 nanosensor in presence and absence of IL-6 in 1x PBS, SWCNT-$(TAT)_6$ (1137 ± 0.6 nm) and Ab IL-6 conjugated (1135 ± 0.75 nm) (difference in means = 3.66 nm, p=0.02, two-tailed t-test).

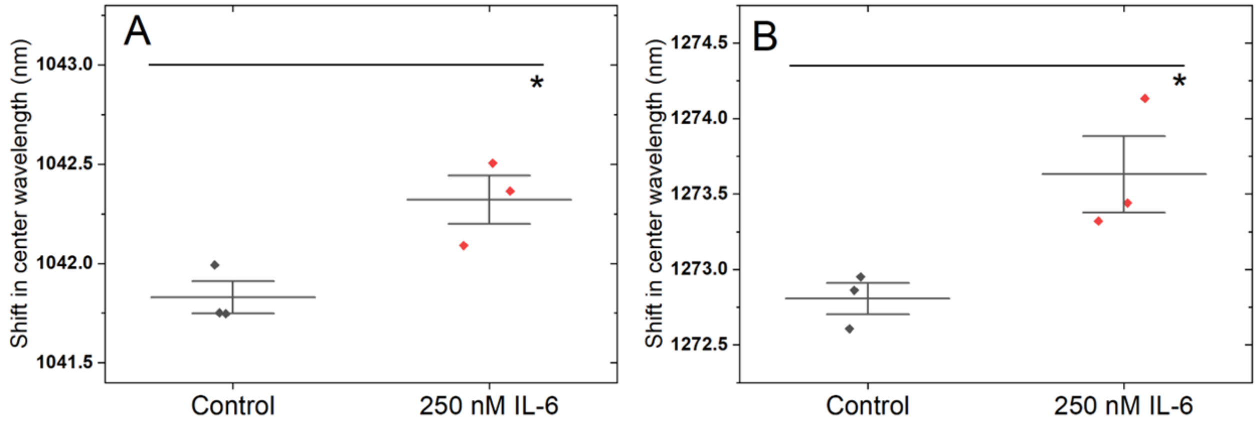

Figure S28: In vitro performance of the IL-6 nanosensor. Performance of the IL-6 nanosensor was assessed by comparing change in the emission peak for IL-6 nanosensor in presence and absence of IL-6 in 1x PBS, (A) for (7,5), SWCNT-$(TAT)_6$ (1041.8 ± 0.14 nm) and Ab IL-6 conjugated (1042.3 ± 0.2 nm) (difference in means = 3.66 nm, p=0.02, two-tailed t-test). (B) For (9,5) SWCNT-$(TAT)_6$ (1272.8 ± 0.18 nm) and Ab IL-6 conjugated (1273.6 ± 0.44 nm) (difference in means = 3 nm, p=0.04, two-tailed t-test)

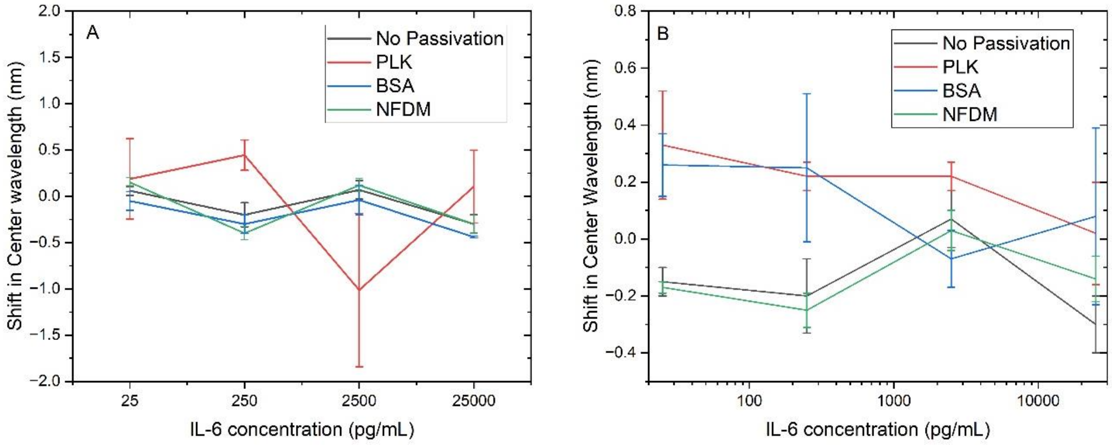

Figure S29: Comparison of response with all passivation agents to IL-6 concentration curve in human serum for additional chiralities. Response to concentration range of IL-6 by (A) (7,6) chirality nanosensor and (B) by (7,5) chirality nanosensor. PLK passivation shows response to all except 25000 pg/mL concentration for (7,6) chirality. PLK as well as BSA passivated (7,5) chirality shows response to IL-6 concentration range.

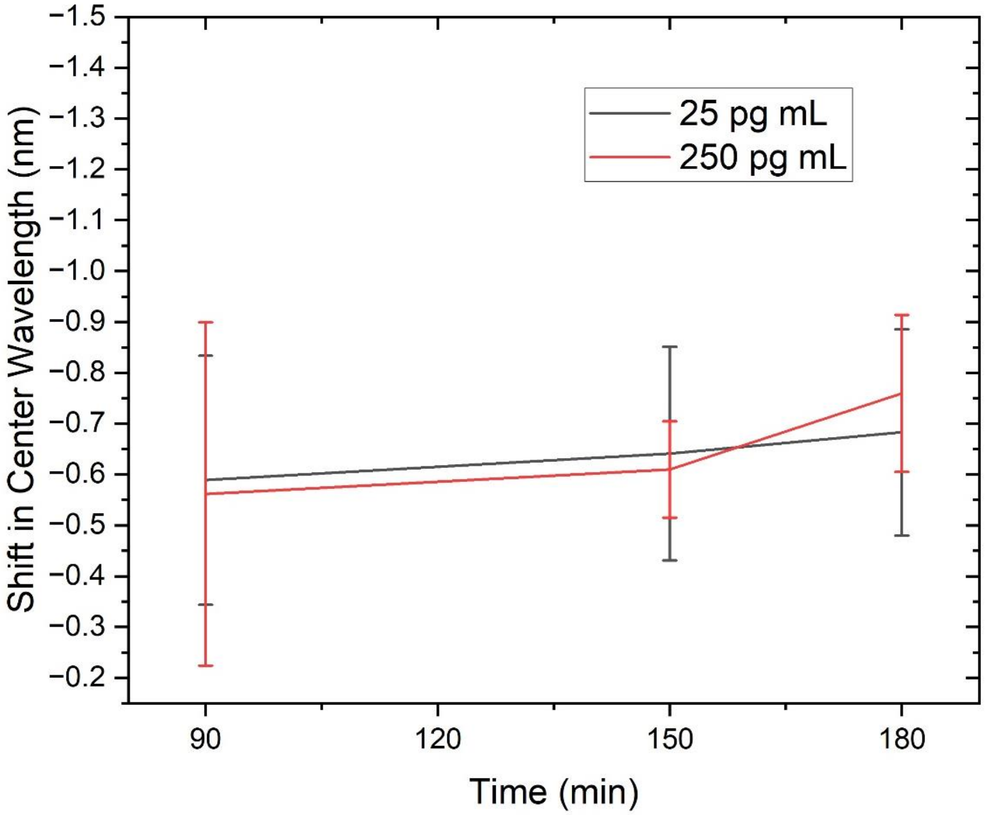

Figure S30: Stability of response over time in human serum. For PLK passivated (8,7) chirality, the lowest IL-6 concentrations, 25 pg/mL and 250 pg/mL show stable response over at least 3 hours, indicating stability of PLK passivation.

	No passivation				Bovine Serum Albumin				Poly-L-Lysine				Non-Fat Dry Milk			
	(7,6)	(7,5)	(9,4)	(8,7)	(7,6)	(7,5)	(9,4)	(8,7)	(7,6)	(7,5)	(9,4)	(8,7)	(7,6)	(7,5)	(9,4)	(8,7)
**25 pg/mL**	0.06 ± 0.05	-0.15 ± 0.05	0.35 ± 0.02	0.18 ± 0.41	-0.05 ± 0.1	0.26 ± 0.11	0.55 ± 0.13 [*]	0.53 ± 0.08 [**]	0.33 ± 0.13	0.33 ± 0.19 [*]	0.1 ± 0.14	-0.68 ± 0.10 [**]	0.15 ± 0.05	-0.17 ± 0.02	0.28 ± 0.02	0.14 ± 0.04
**250 pg/mL**	-0.2 ± 0.13	-0.01 ± 0.3	-0.02 ± 0.17	-0.2 ± 0.3	-0.3 ± 0.1	0.25 ± 0.26	0.36 ± 0.11 [**]	0.25 ± 0.11	0.42 ± 0.11 [***]	0.22 ± 0.05	-0.11 0.03 [**]	-0.76 ± 0.08 [***]	-0.4 ± 0.07	-0.25 ± 0.06	-0.07 ± 0.03	-0.15 ± 0.08
**2,500 pg/mL**	0.07 ± 0.1	-0.2 ± 0.06	0.35 ± 0.13	0.32 ± 0.41	-0.04 ± 0.16	-0.07 ± 0.1	0.29 ± 0.08 [*]	0.41 ± 0.03 [***]	0.8 ± 0.43 [*]	0.22 ± 0.05 [**]	-0.15 ± 0.09	-0.62 ± 0.32	0.12 ± 0.07	0.03 ± 0.07	0.2 ± 0.1	0.07 ± 0.03
**25,000 pg/mL**	-0.3 ± 0.1	-0.05 ± 0.04	0.03 ± 0.05	0.19 ± 0.43	-0.44 ± 0.01	0.08 ± 0.31	0.04 ± 0.05	0.09 ± 0.09	-0.07 ± 0.6	0.02 ± 0.18	-0.32 ± 0.03 [***]	-0.94 ± 0.18 [**]	-0.3 ± 0.1	-0.14 ± 0.08	-0.005 ± 0.11	0.05 ± 0.04

Figure S31. Detection of IL-6 in human serum. Shifts in center wavelength (mean ± standard deviation) are indicated for each of the (7,6), (7,5), (9,4), and (8,7) SWCNT chiralities after addition of the indicated concentration of IL-6, categorized by passivation agent (note: a "-" indicates blue shift). Light grey indicates non-passivated controls and dark grey indicates changes which are not statistically significant. Blue indicates statistically significant blue shifts, while red indicates statistically significant red shifts. * = $p < 0.05$; ** = $p < 0.01$; *** = $p < 0.001$).

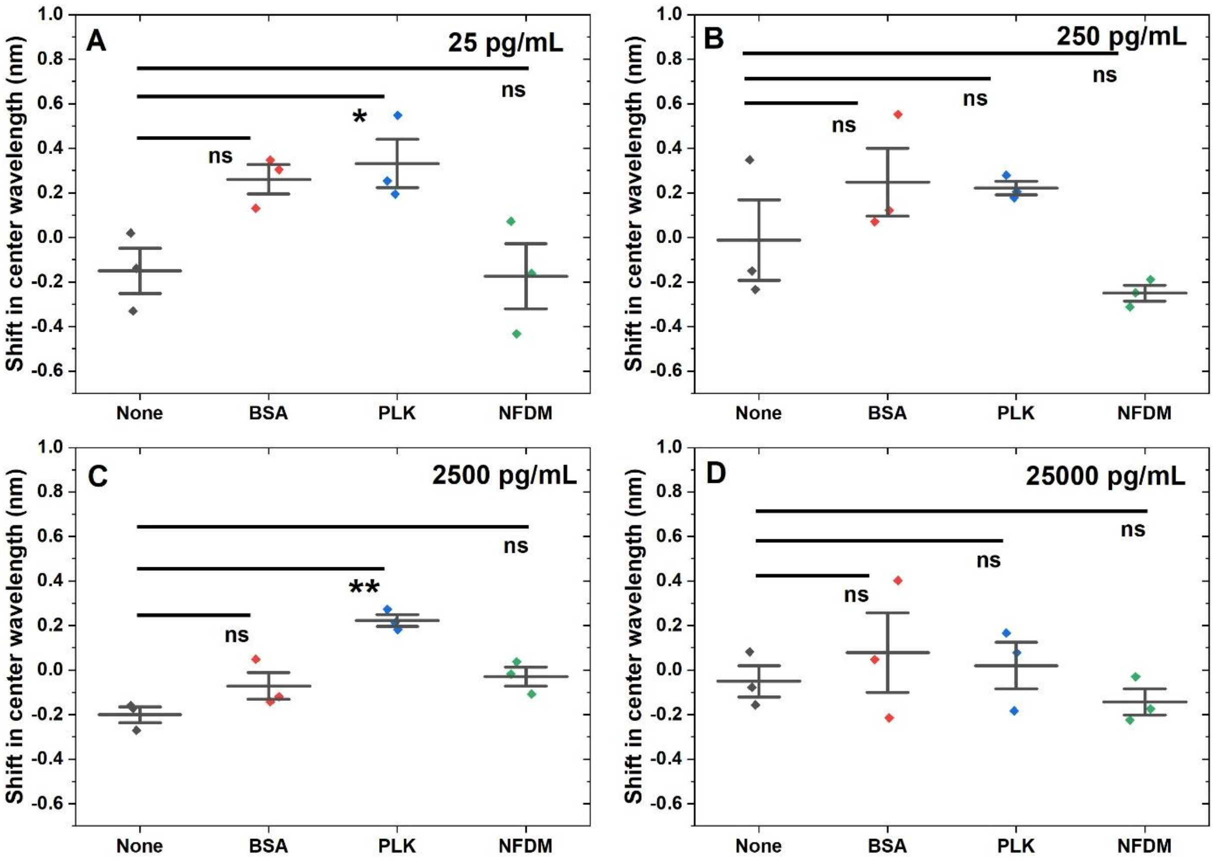

Figure S32. Response of the (7,5) nanosensor chirality to IL-6 in human serum. Shift in emission center wavelength for (7,5) chirality of IL-6 nanosensor to (A) 25 pg/mL IL-6. n=3, shows detection only upon PLK passivation. (B) Nanosensor response to 250 pg/mL IL-6. n=3, is not significant in case of all passivations. (C) Nanosensor response to 2,500 pg/mL IL-6. n=3, (D) Nanosensor does not respond to 25,000 pg/mL IL-6 n=3 following any passivation.

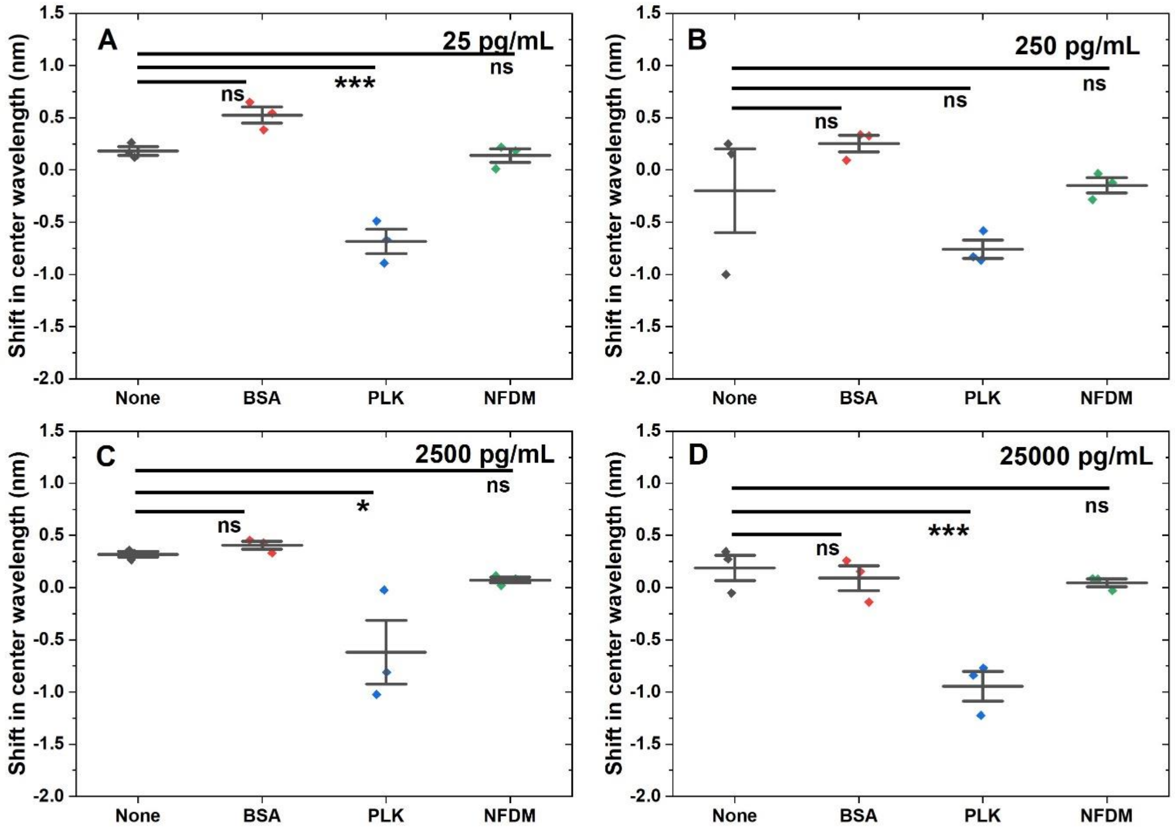

Figure S33. Response of the (8,7) nanosensor chirality to IL-6 in human serum. Shift in emission center wavelength for (8,7) chirality of the IL-6 nanosensor in response to (A) 25 pg/mL IL-6. n=3,is significant for PLK passivation indicating successful detection. (B) Nanosensor does not detect 250 pg/mL IL-6. n=3, upon any passivation conditions. (C) Nanosensor detects 2,500 pg/mL IL-6. n=3, upon PLK passivation only. (D) Nanosensor detects 25,000 pg/mL IL-6. n=3, upon PLK passivation only.

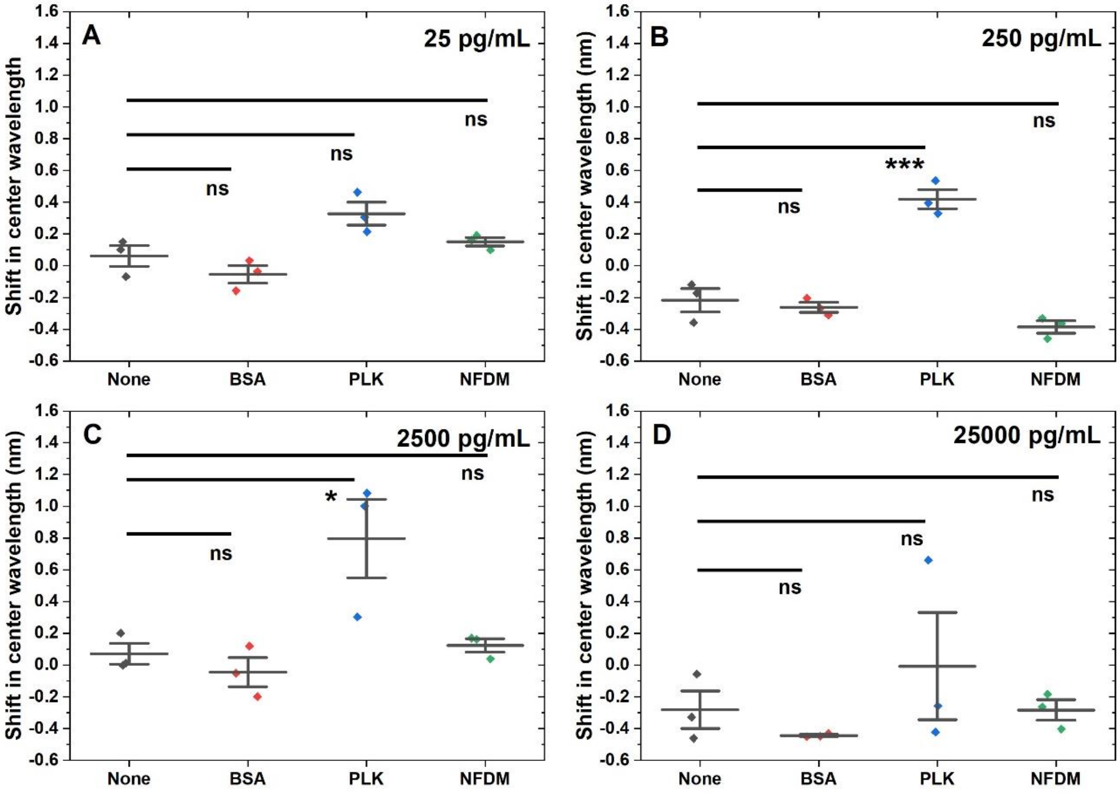

Figure S34. Response of the (7,6) nanosensor chirality to IL-6 in human serum. Shift in emission center wavelength for (7,6) chirality of the IL-6 nanosensor in response to (A) 25 pg/mL IL-6. n=3, does not show detection for any passivation agents. The nanosensor detects (B) 250 pg/mL IL-6. N=3 and (C) 2500 pg/mL IL-6. n=3, only upon PLK passivation. (D) nanosensor does not detect 25,000 pg/mL IL-6. n=3,

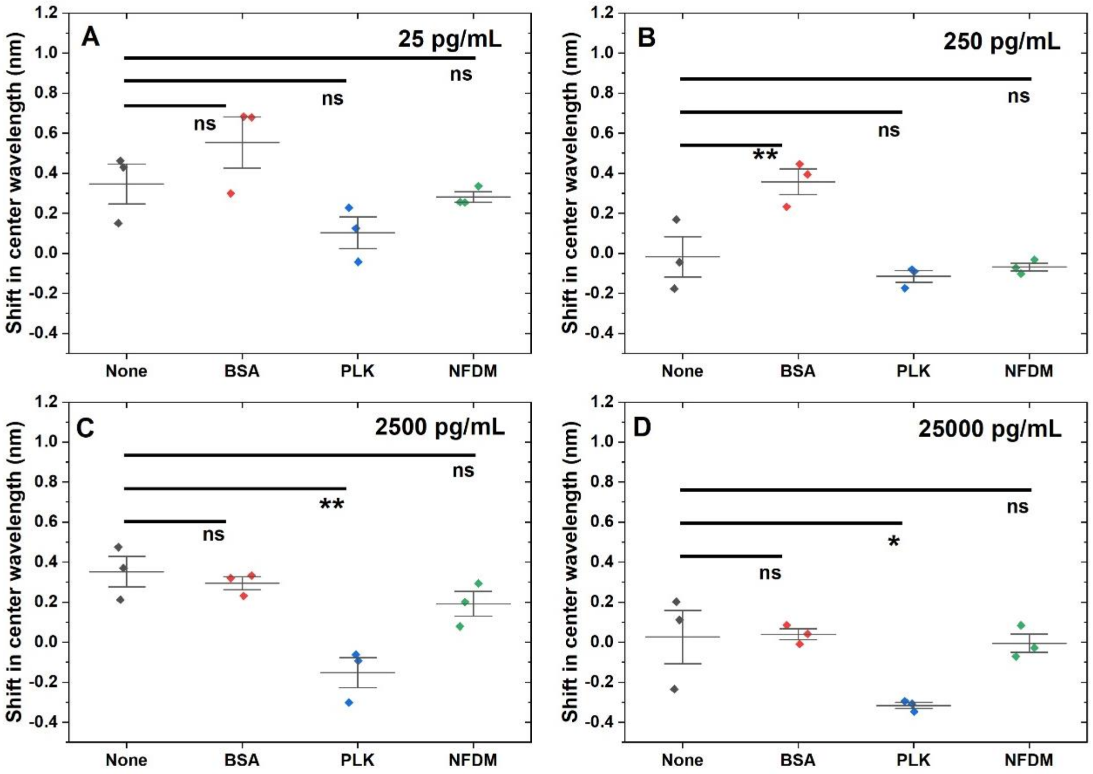

Figure S35. Response of the (9,4) nanosensor chirality to IL-6 in human serum. Shift in emission center wavelength for (9,4) chirality of the IL-6 nanosensor in response to (A) 25 pg/mL IL-6. n=3, does not show detection for any passivations. d(B) Nanosensor detects 250 pg/mL IL-6. n=3, only upon BSA passivation. (C) Nanosensor detects 2,500 pg/mL IL-6. n=3, for PLK passivation only. (D) The nanosensor detects 25,000 pg/mL IL-6. n=3, upon PLK passivation only.
